# Comparison of direct and inverse methods for 2.5D traction force microscopy

**DOI:** 10.1371/journal.pone.0262773

**Published:** 2022-01-20

**Authors:** Johannes W. Blumberg, Ulrich S. Schwarz

**Affiliations:** Heidelberg University, Institute for Theoretical Physics and Bioquant, Heidelberg, Germany; University of Zaragoza, SPAIN

## Abstract

Essential cellular processes such as cell adhesion, migration and division strongly depend on mechanical forces. The standard method to measure cell forces is traction force microscopy (TFM) on soft elastic substrates with embedded marker beads. While in 2D TFM one only reconstructs tangential forces, in 2.5D TFM one also considers normal forces. Here we present a systematic comparison between two fundamentally different approaches to 2.5D TFM, which in particular require different methods to deal with noise in the displacement data. In the direct method, one calculates strain and stress tensors directly from the displacement data, which in principle requires a divergence correction. In the inverse method, one minimizes the difference between estimated and measured displacements, which requires some kind of regularization. By calculating the required Green’s functions in Fourier space from Boussinesq-Cerruti potential functions, we first derive a new variant of 2.5D Fourier Transform Traction Cytometry (FTTC). To simulate realistic traction patterns, we make use of an analytical solution for Hertz-like adhesion patches. We find that FTTC works best if only tangential forces are reconstructed, that 2.5D FTTC is more precise for small noise, but that the performance of the direct method approaches the one of 2.5D FTTC for larger noise, before both fail for very large noise. Moreover we find that a divergence correction is not really needed for the direct method and that it profits more from increased resolution than the inverse method.

## Introduction

Mechanical forces are important for a wide range of essential cellular processes such as cell adhesion, migration and division [1]. The new field of mechanobiology has evolved around the role of forces in biological systems [2]. However, in general forces cannot be observed directly, but have to be measured by their effect to create deformation and flow. The key idea to measure cell forces therefore is to use a calibrated material as a measurement devise. The simplest approach is to replace the glass or plastic substrates commonly used for cell culture by soft elastic substrates and to measure their deformations by tracking embedded marker beads. This method is known as traction force microscopy (*TFM*) and is widely used in cell biology and biophysics [3–8]. Depending on whether one aims at reconstructing only tangential or also normal forces, one speaks of *2D TFM* or *2.5D TFM*, respectively [8]. If one goes beyond planar substrates and reconstructs cell forces applied to a fibrous marix surrounding the cell, then one speaks of *3D TFM* [7–10]. Recently the concept of measuring cell forces on the surface of calibrated material has been extended beyond planar substrates by using elastic beads deformed by cell traction [11–13].

Recalling the concepts of continuum mechanics, in principle it is straight-forward to calculate cell traction from displacement data. Given the three-dimensional displacement field, one can first calculate the strain tensor, which is its derivative. Using the constitutive law of the material, it can be converted into a stress tensor. For linear isotropic material, this is simply a linear transformation. Finally the surface traction follows as contraction of the stress tensor with the surface normal. This workflow is known as the *direct* (or *forward*) *method* (DM). It has been pioneered for 2.5D TFM [14–16], but also has been applied to 3D TFM [17, 18] and to elastic beads with embedded markers that are deformed by the traction forces in cell aggregates [11]. The DM is especially suited to deal with large deformations and non-linear material laws, but it also can be used in the linear regime, which is typically used for 2D or 2.5D TFM on soft elastic substrates. However, its use is not very common in this field, for two main reasons. First the DM requires a 3D image volume in order to be able to reconstruct the full stress tensor, which is challenging given the anisotropic point spread functions of standard microscopes and the time required to acquire image stacks. Planar substrates are more compatible with imaging in 2D focal planes rather than 3D image volumes. Secondly, it is a numerical challenge to calculate the required derivatives in the presence of the unavoidable experimental noise, especially when microscope resolution is low.

Rather than using the direct method, on soft elastic substrates it is common practice to use the *inverse method* that was conceived by the pioneers of 2D TFM [19, 20]. In order to avoid derivatives, one does not explicitly calculate strain or stress tensors, but stays on the level of displacement fields. By minimizing the difference between the measured displacement field and a displacement field calculated from an estimated traction field, one arrives at the best estimate given the experimental data. Because elasticity theory leads to an ill-posed inverse problem due to its long-ranged deformation fields, one usually deals with the noise problem by invoking some regularization procedure, e.g. zero-order Tikhonov regularization [4, 8, 20, 21]. In order to choose the correct value of the regularization parameter, different schemes have been suggested, including the L-curve criterion or generalized cross-validation [22, 23]. The need for explicit regularization can be avoided by using TFM-schemes that effectively filter the deformation data, like image smoothing [24, 25] or the Finite Element Method (FEM) [17, 18, 26].

While the inverse method avoids the calculation of derivatives, it requires to repeatedly calculate deformation from estimated traction (*direct problem*). This can be implemented with different methods, but the two most common ones are Green’s functions (GFs) and FEM. GFs can be used only for linear elasticity, but offer several advantages. First, the GF for thick elastic substrates is well known (*Boussinesq solution*) [27] and also the GF for a finite thickness substrate has been derived [28]. Second, for flat cells on planar substrates it is sufficient to know the displacements in a two-dimensional plane close to the gel surface. This reduces the Green’s function from a 3x3 to a 2x2 matrix. And third, one can use fast Fourier methods to convert the convolution of GF and traction into a product. Due to its speed, Fourier Transform Traction Cytometry (FTTC) [24] therefore has become the method of choice for high resolution measurements. Moreover regularization can be formulated in Fourier space and therefore many schemes can be applied to deal with the noise issue [23, 29, 30]. Together, these advances make the inverse method very attractive for measuring cell traction on soft elastic substrates. If cell forces in the third dimension are also of interest (*2.5D TFM*), one often switches to implementations that use FEM to solve the direct problem [31]. Alternatively, an analytical scheme has been proposed for 2.5D FTTC before, which however is rather complicated because it has been derived for substrates with finite thickness [28].

In summary, TFM has become a very diverse field with a mix of different approaches, each of which have their advantages and disadvantages in a certain context. Very rarely however are these different methods directly compared against each other. A notable exception is a recent work that compares FEM-based implementations of the direct and inverse methods for 3D TFM [17, 18]. Here we aim at a similar comparison, but for 2.5D TFM and with GF-based methods. Rather than simulating experimental setups, we use simple test cases and simulations with displacement noise to provide a comprehensive comparison of the mathematical properties of direct versus inverse methods for 2.5D TFM.

In addition to this interest in fundamental questions of TFM, our work is also motivated by different recent experimental developments. First, the DM seems to be an attractive choice for non-planar geometries like elastic beads, for which it is very challenging to calculate appropriate GFs [11]. We expect that this line of research will become more important in the future with the promise of additive manufacturing to print 3D elastic material that is compatible with cell culture and deforms under cell traction [32–35]. Such systems might by approached best with FEM-approaches, but in some cases (like elastic beads) GF-based inverse methods are possible [12, 36]. Here we use this recent development as a motivation to compare direct and inverse methods in the traditional setup of 2.5D TFM for planar substrates.

Second, new microscopy methods have been used to achieve better image resolution for 2.5D TFM, including Stimulated Emission Depletion (STED) microscopy [37], Structured Illumination Microscopy (SIM) [26, 38], astigmatic SIM [39] and fluctuation-based TFM [40]. The displacement data resulting from these experimental advances is often analyzed with one of the different TFM-methods, but a systematic comparison between different methods is usually not performed. In this context, it is interesting to know how direct and inverse methods compare in regard to improvements in sampling density. Here we use this recent development as a motivation to also study the effect of varying sampling distance.

Our work is structured as follows. We first provide an in-depth introduction to direct and inverse methods for 2.5D TFM. We use Boussinesq-Cerruti potential functions to derive a novel and very efficient version of the inverse method 2.5D FTTC. For the 2.5D DM, we discuss different schemes for numerically calculating the required derivatives and introduce a divergence correction motivated by similar schemes from hydrodynamics. We define several simple test cases for force reconstruction based on analytical solutions for Hertz-like adhesion patches. To test the robustness of our different methods, we simulate different levels of displacement noise, which is a common method to lump noise that might originate from optical microscopy or gel preparation into one parameter. In addition, we investigate the effect of varying sampling distance. Our results show that 2.5D FTTC usually performs better than the 2.5D DM, but that the performance of the DM approaches the one of FTTC for larger noise, before both fail at very large noise. We also find that the divergence correction for the DM is not needed and that DM profits more from increased sampling density.

## Theory and methods

### Inverse versus direct methods

**Fig 1:**
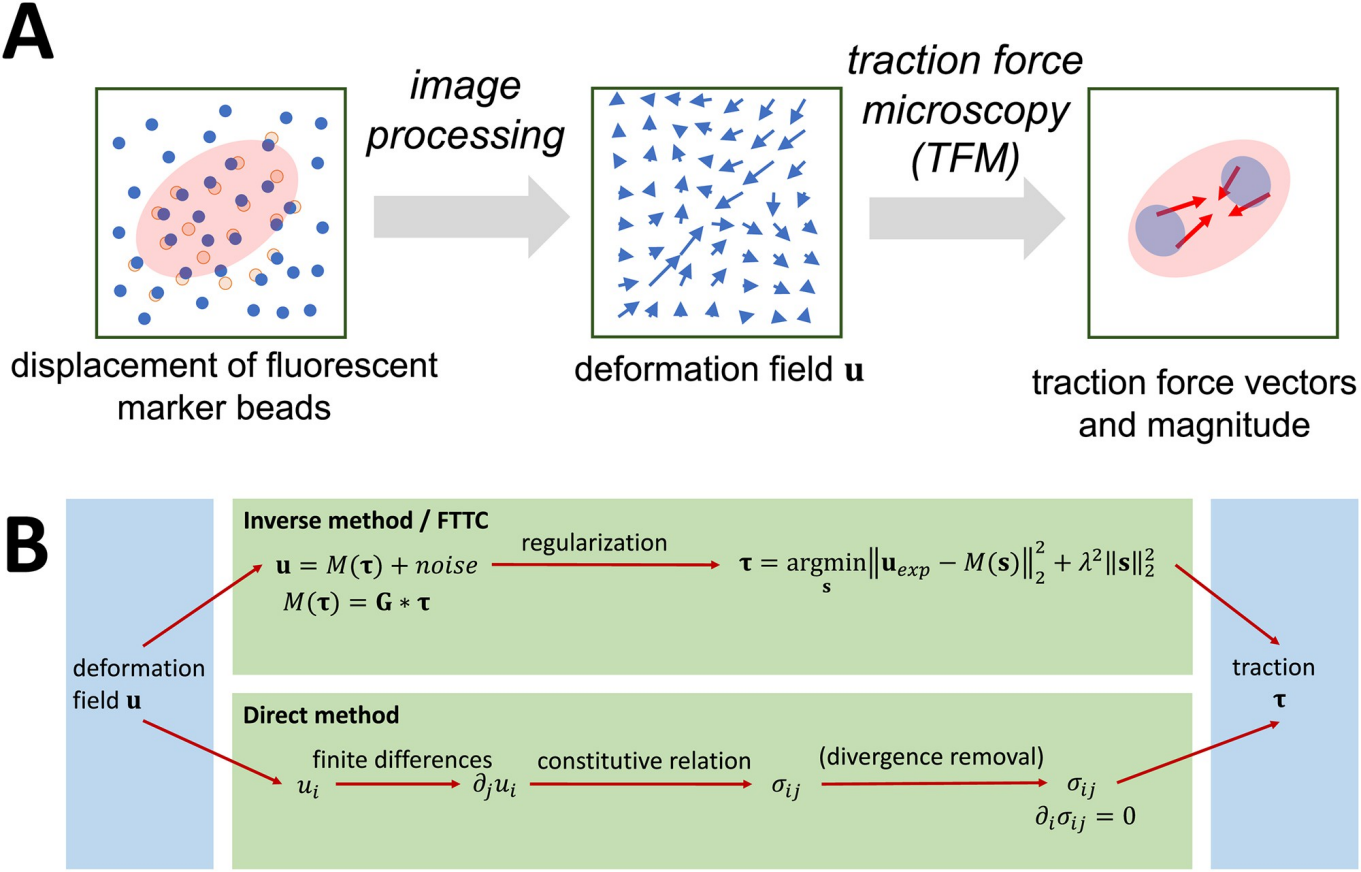
General overview of traction force microscopy. (A) By determining with image processing the movement of marker beads in the substrate due to cell forces, a deformation field is obtained. Traction force microscopy (TFM) estimates the cellular traction field from the displacement data. (B) Here we discuss two fundamentally different methods for TFM. The inverse method estimates a force distribution that results in an optimal match for the global displacement field. In Fourier Transform Traction Cytometry (FTTC) one makes use of fast Fourier transforms. A regularization scheme is introduced to address the fact that the inverse problem is ill-posed. In the direct method, point-wise computational methods are used to determine the stress tensor locally. A divergence correction can be applied to ensure that the physical force balance ∂_*i*_ *σ*_*ij*_ = 0 is satisfied.

The standard workflow of a TFM analysis for planar elastic substrates is outlined in Fig 1A. Deformations are usually determined by comparing the situation in which the cell has fully adhered to the substrate with a reference situation in which the substrate is relaxed and the cells exert no forces, e.g. by removing or relaxing the cell. The state of the substrate can also be observed at multiple time steps to observe the temporal evolution of the cell-to-substrate adhesion [41]. Deformations in the substrate are commonly monitored with the help of fluorescent marker beads, whose change in position can be observed in the microscope. The bead displacement is obtained using common tracking and correlation techniques [3, 4, 42]. Next, traction force routines are employed to estimate the traction force vector field from the displacement vector field. Here we compare two methods as shown in Fig 1B. The traditional approach introduced by the pioneers of 2D TFM is the inverse method (top), which minimizes the difference between the experimental and estimated displacement fields. The direct method (bottom) cannot be used in a purely 2D setup, but requires 3D image data. In this method, traction is calculated directly from the displacement field by differentiation and (linear) transformation between strain and stress. For both methods, elements of elasticity theory are required.

### Elasticity theory

Traction forces are forces acting on the boundary of elastic solids. They are quantified by a three-component vector field ***τ***, which is defined for all points at a surface and describes the area density of the force. Its component normal to the surface describes the pressure balance between the solid and its surroundings. In contrast, the tangential components describe shear forces.

The stress tensor ***σ*** describes the force per area acting on any (real or fictitious) surface of the system. It is defined by the relation:

$$\begin{array}{c} \tau_{S}=\sigma n_{S}\;. \end{array}$$ (1)

Here, the left hand side represents the surface force density for a given surface S. The vector $n_{S}$ is the unit normal vector of S. In the case of a solid which is delimited by a planar surface at the *z* = 0 plane and whose outwards normal is defined in negative z-direction, the traction stress reads:

$$\begin{array}{c} \tau =-(\sigma_{13},\sigma_{23},\sigma_{33}){|}_{z=0}\;. \end{array}$$ (2)

This means that by determining the stress tensor in a region close to the surface, the surface traction is known.

The different entries to the stress tensor are not independent physical quantities, because they have to obey force and torque balances. The stresses lead to material movement that is described by the deformation gradient tensor **F**, which is the Jacobian of the coordinate transformation between deformed and undeformed configurations. Alternatively the movement can be described by the deformation field **u**, which is related to the deformation gradient tensor by

$$\begin{array}{c} F_{ij}=\delta_{ij}+\frac{\partial u_{i}}{\partial x_{j}}. \end{array}$$ (3)

Note that the derivatives must be taken with respect to the coordinates of the reference configuration (*Lagrange frame*).

Stresses within the material and changes in its configuration are related by a constitutive equation ***σ*** = ***σ***(**F**), which is specific to the material in question (*material law*). For relatively stiff homogeneous and isotropic materials, a linear approximation can be used that is given by

$$\begin{array}{c} \sigma_{ij}=\frac{E}{2(1+\nu )}(F_{ij}+F_{ji}+\frac{2\nu (\text{tr}(F)-1)-2}{1-2\nu }\delta_{ij})\; \end{array}$$ (4)

or in terms of the displacement field

$$\begin{array}{c} \sigma_{ij}=\frac{E}{2(1+\nu )}(\frac{\partial u_{i}}{\partial x_{j}}+\frac{\partial u_{i}}{\partial x_{j}}+\frac{2\nu }{1-2\nu }\left(\nabla u\right)\delta_{ij})\;. \end{array}$$ (5)

The material constants *E* and *ν* are known as the Young’s modulus and the Poisson’s ratio, respectively, and describe the stiffness and compressibility of the material. Note that the difference between the coordinates of the reference configuration (Lagrange frame) and the coordinates describing positions in the Euclidean space (Euler frame) are neglected in this linear approximation.

Whether a substrate is considered stiff or soft is determined by the relation between the typical traction force amplitude and the Young’s modulus. The dimensionless deformation gradient will typically be in the same magnitude as the ratio *σ*_*ij*_/*E*. We must consider the substrate to be soft if we expect the magnitude of the traction forces to be much larger than the substrates Young’s modulus. Then the linear approximation as given here does not necessarily apply anymore and might has to be replaced by a more complicated (non-linear) mapping. Typical substrate materials used for TFM are polyacrylamide (PAA) or polydimethlysiloxane (PDMS), that can be created for a wide range of stiffnesses. For TFM-experiments, a typical value is 10 kpa, which according to the above considerations is a rather stiff substrates assuming typical traction stresses in the range of 1 kpa. Therefore the linear relation is typically satisfied. Both types of substrates are usually considered to be close to incompressible, with a Poisson ratio close to 1/2. It should be noted that incompressibility implies ∇**u** = 0 in the linear case assumed in Eq (5). This counteracts the apparent divergence for *ν* → 1/2 that would otherwise occur in the last term of Eq (5).

Assuming small deformations and a linear material law, the traction forces can be related to deformations directly using a convolution relation:

$$\begin{array}{c} u\left(x,y,z\right)=\int_{S}G\left(x-x^{\prime },y-y^{\prime },z\right)\cdot \tau \left(x^{\prime },y^{\prime }\right)\text{d}x^{\prime }\;\text{d}y^{\prime }, \end{array}$$ (6)

where the coordinate system is chosen in such a way that the traction stresses are exerted on the *z* = 0 plane and the substrate is confined to the *z* > 0 halfspace. The GF **G** is known analytically for an elastic halfspace (*Boussinesq solution*) [27]:

$$\begin{array}{c} \begin{array}{cc} & \frac{2\pi E}{1+\nu }G\left(x,y,z\right)=\\ & \left(\begin{array}{ccc} \frac{2(1-\nu )r+z}{r(r+z)}+\frac{\left(2r\left(\nu r+z\right)+z^{2}\right)x^{2}}{r^{3}{\left(r+z\right)}^{2}} & \frac{(2r\left(\nu r+z\right)+z^{2})xy}{r^{3}{\left(r+z\right)}^{2}} & \frac{xz}{r^{3}}-\frac{(1-2\nu )x}{r(r+z)}\\ \frac{(2r\left(\nu r+z\right)+z^{2})xy}{r^{3}{\left(r+z\right)}^{2}} & \frac{2(1-\nu )r+z}{r(r+z)}+\frac{\left(2r\left(\nu r+z\right)+z^{2}\right)y^{2}}{r^{3}{\left(r+z\right)}^{2}} & \frac{yz}{r^{3}}-\frac{(1-2\nu )y}{r(r+z)}\\ \frac{(1-2\nu )x}{r(r+z)}+\frac{xz}{r^{3}} & \frac{(1-2\nu )y}{r(r+z)}+\frac{yz}{r^{3}} & \frac{2(1-\nu )}{r}+\frac{z^{2}}{r^{3}} \end{array}\right) \end{array}, \end{array}$$ (7)

where we used $r=\sqrt{x^{2}+y^{2}+z^{2}}$. Cases where the substrate is not sufficiently thick and interactions at the bottom surface of a finite-thickness substrate can not be neglected have also been studied [28]. In these cases the convolution relation is still valid, but a different GF has to be used. Note that the 1/*r*-dependence of this GF is the reason why the inverse problem is ill-posed.

### 2.5D Fourier Transform Traction Cytometry (FTTC)

FTTC is the most widely used inverse method and uses the fact that the convolution integral from Eq (6) becomes a product in Fourier space and that fast Fourier transforms allow one to quickly switch between real and Fourier space [24, 29, 37, 43]. To use it, the displacement field **u**(**x**) must first be interpolated onto a regular, rectangular grid covering the whole image, either on the surface or in a fixed depth *z* = *h* within the substrate. We will designate the values at the sample points **x**_*ij*_ by **u**_*ij*_ in the following. Then the traction force ***τ***(*x*, *y*) is described by a set of plane waves ${\hat{f}}_{mn}\left(x,y\right)$

$$\begin{array}{cccc} \tau (x,y) & =\sum_{m=0}^{N_{x}}\sum_{n=0}^{N_{y}}{\hat{\tau }}_{mn}{\hat{f}}_{mn}\left(x,y\right)\;, & {\hat{f}}_{mn}\left(x\right) & =\frac{1}{N_{x}N_{y}}e^{ik_{mn}\cdot x}\;. \end{array}$$ (8)

The wave vectors **k**_*mn*_ are chosen in accordance with the sampling grid. This choice ensures that the expansion coefficients ${\hat{\tau }}_{mn}$ are in fact the 2D discrete Fourier transform of the traction sampled at the **x**_*ij*_. Due to the Fourier convolution theorem, Eq (6) reduces to

$$\begin{array}{c} {\hat{u}}_{mn}=\tilde{G}({\left(k_{mn}\right)}_{x},{\left(k_{mn}\right)}_{y},h)\cdot {\hat{\tau }}_{mn}, \end{array}$$ (9)

where ${\hat{u}}_{mn}$ and **u**_*ij*_ are also related by a 2D discrete Fourier transform. The term $\tilde{G}\left(k_{x},k_{y},z\right)$ describes the function obtained from applying a continuous Fourier transform in the two tangential directions on the real space GF.

For 2D FTTC, one uses only the planar part at *z* = 0 [4, 21, 23, 24]:

$$\begin{array}{c} {\tilde{G}}_{2D}\left(k_{x},k_{y}\right)=\frac{1}{\mu k^{3}}(\begin{array}{cc} \left(1-\nu \right)k_{x}^{2}+\nu k_{y}^{2} & -\nu k_{x}k_{y}\\ -\nu k_{x}k_{y} & \left(1-\nu \right)k_{y}^{2}+\nu k_{x}^{2} \end{array}). \end{array}$$ (10)

Here we used $k=\sqrt{k_{x}^{2}+k_{y}^{2}}$ and *μ* = *E*/2(1 + *ν*). Note that this result differs in an important minus sign in the off-diagonal elements from the one given in the original publication on FTTC [24].

2.5D FTTC was first introduced by [28] for thin substrates using a rather lengthy expression to derive a stress-strain relation. Here, we assume a thick substrate and present a more straight forward calculation to derive a closed form solution for the GF in Fourier spaces, that is consistent with the 2D formula presented in (10) and effectively gives the same results as the procedure given earlier in the context of a finite thickness halfspace [28].

The general problem of finding the deformation field in an infinite halfspace with surface traction as boundary condition and no internal forces follows from Eq 5 as

$$\begin{array}{c} \frac{\partial }{\partial x_{j}}\left(\frac{\partial u_{i}}{\partial x_{j}}+\frac{\partial u_{j}}{\partial x_{i}}+\frac{2\nu }{1-2\nu }\left(\nabla u\right)\delta_{ij}\right)=0,-\sigma_{iz}{|}_{z=0}=\tau_{i}. \end{array}$$ (11)

The GF in real space is known as described by Eq 6 with the Boussinesq equation [27]. By making use of the convolution theorem, Eq 6 can be reduced into the simple product expression

$$\begin{array}{c} {\tilde{u}}_{i}\left(k_{x},k_{y},z\right)=\sum_{j=1}^{3}{\tilde{G}}_{ij}\left(k_{x},k_{y},z\right){\tilde{q}}_{j}\left(k_{x},k_{y}\right). \end{array}$$ (12)

Here a tilde above the quantities represents the Fourier transform along the *x* and *y* axis. Unfortunately, finding an analytical expression for ${\tilde{G}}_{ij}$ directly from transforming the real space Boussinesq solution would require us to solve a complex integral expression. Instead of doing so, we will derive the relation between $\tilde{\tau }$ and $\tilde{u}$ directly from the boundary problem stated in Eq 11. We can then extract the analytical expression for ${\tilde{G}}_{ij}$ by comparing this relation to Eq 12. In general, our procedure follows the same steps as described in Ref [27] to derive the real space GF in the first place.

We start by introducing a set of harmonic potential functions that solve the boundary value problem stated by:

$$\begin{array}{c} \nabla^{2}P_{i}=0\;,\;\partial_{z}^{3}P_{i}{|}_{z=0}=-\tau_{i}\;. \end{array}$$ (13)

These Boussinesq-Cerruti potential functions have been described e.g. in Refs. [44, 45]. A solution of Eq (11) is now given by

$$\begin{array}{c} u_{i}=\sum_{j=1}^{3}u_{i}^{\left(j\right)}, \end{array}$$ (14)

together with the *x*-tangential contributions

$$\begin{array}{c} \begin{array}{cc} 2\mu u_{x}^{\left(x\right)} & =2\nu \partial_{x}^{2}P_{x}+2\partial_{z}^{2}P_{x}-z\partial_{x}^{2}\partial_{z}P_{x}\\ 2\mu u_{y}^{\left(x\right)} & =2\nu \partial_{x}\partial_{y}P_{x}-z\partial_{x}\partial_{y}\partial_{z}P_{x}\\ 2\mu u_{z}^{\left(x\right)} & =\left(1-2\nu \right)\partial_{x}\partial_{z}P_{x}-z\partial_{x}\partial_{z}^{2}P_{x}, \end{array} \end{array}$$ (15)

the *y*-tangential contributions

$$\begin{array}{c} \begin{array}{cc} 2\mu u_{x}^{\left(y\right)} & =2\nu \partial_{x}\partial_{y}P_{y}-z\partial_{x}\partial_{y}\partial_{z}P_{y}\\ 2\mu u_{y}^{\left(y\right)} & =2\nu \partial_{y}^{2}P_{y}+2\partial_{z}^{2}P_{y}-z\partial_{y}^{2}\partial_{z}P_{y}\\ 2\mu u_{z}^{\left(y\right)} & =\left(1-2\nu \right)\partial_{y}\partial_{z}P_{y}-z\partial_{y}\partial_{z}^{2}P_{y} \end{array} \end{array}$$ (16)

and the normal (*z*) contributions

$$\begin{array}{c} \begin{array}{cc} 2\mu u_{x}^{\left(z\right)} & =-\left(1-2\nu \right)\partial_{x}\partial_{z}P_{z}-z\partial_{x}\partial_{z}^{2}P_{z}\\ 2\mu u_{y}^{\left(z\right)} & =-\left(1-2\nu \right)\partial_{y}\partial_{z}P_{z}-z\partial_{y}\partial_{z}^{2}P_{z}\\ 2\mu u_{z}^{\left(z\right)} & =2\left(1-\nu \right)\partial_{z}^{2}P_{z}-z\partial_{z}^{3}P_{z}. \end{array} \end{array}$$ (17)

By applying the mentioned two-dimensional Fourier transform to Eq (13), the different modes decouple and we obtain the following initial value problem:

$$\begin{array}{c} \partial_{z}^{2}{\tilde{P}}_{i}\left(k_{x},k_{y},z\right)-\left(k_{x}^{2}+k_{y}^{2}\right){\tilde{P}}_{i}\left(k_{x},k_{y},z\right)=0\;,\;\partial_{z}^{3}{\tilde{P}}_{i}{|}_{z=0}=-{\tilde{\tau }}_{i}\;. \end{array}$$ (18)

Because the differential equation is linear, it can easily be solved and we find for the Fourier transform of the potential functions:

$$\begin{array}{cc} {\tilde{P}}_{i}\left(k_{x},k_{y},z\right) & =\frac{1}{{\sqrt{k_{x}^{2}+k_{y}^{2}}}^{3}}e^{-\sqrt{k_{x}^{2}+k_{y}^{2}}z}{\tilde{\tau }}_{i}\left(k_{x},k_{y}\right). \end{array}$$ (19)

In the next step, we apply the Fourier transform onto Eqs (14) to (17) and obtain:

$$\begin{array}{c} {\tilde{u}}_{i}=\sum_{j=1}^{3}{\tilde{u}}_{i}^{\left(j\right)}, \end{array}$$ (20)

together with the *x*-tangential contributions

$$\begin{array}{c} \begin{array}{cc} 2\mu {\tilde{u}}_{x}^{\left(x\right)} & =-2\nu k_{x}^{2}P_{x}+2\partial_{z}^{2}P_{x}+k_{x}^{2}z\partial_{z}P_{x}\\ 2\mu {\tilde{u}}_{y}^{\left(x\right)} & =-2\nu k_{x}k_{y}P_{x}+k_{x}k_{y}z\partial_{z}P_{x}\\ 2\mu {\tilde{u}}_{z}^{\left(x\right)} & =ik_{x}\left(1-2\nu \right)\partial_{z}P_{x}-ik_{x}z\partial_{z}^{2}P_{x}, \end{array} \end{array}$$ (21)

the *y*-tangential contributions

$$\begin{array}{c} \begin{array}{cc} 2\mu {\tilde{u}}_{x}^{\left(y\right)} & =-2\nu k_{x}k_{y}P_{y}-z\partial_{z}P_{y}\\ 2\mu {\tilde{u}}_{y}^{\left(y\right)} & =-2\nu k_{y}^{2}P_{y}+2\partial_{z}^{2}P_{y}+k_{y}^{2}z\partial_{z}P_{y}\\ 2\mu {\tilde{u}}_{z}^{\left(y\right)} & =ik_{x}\left(1-2\nu \right)\partial_{z}P_{y}-ik_{x}z\partial_{z}^{2}P_{y} \end{array} \end{array}$$ (22)

and the normal (*z*) contributions

$$\begin{array}{c} \begin{array}{cc} 2\mu {\tilde{u}}_{x}^{\left(z\right)} & =-\left(1-2\nu \right)ik_{x}\partial_{z}P_{z}-ik_{x}z\partial_{z}^{2}P_{z}\\ 2\mu {\tilde{u}}_{y}^{\left(z\right)} & =-\left(1-2\nu \right)ik_{y}\partial_{z}P_{z}-ik_{y}z\partial_{z}^{2}P_{z}\\ 2\mu {\tilde{u}}_{z}^{\left(z\right)} & =2\left(1-\nu \right)\partial_{z}^{2}P_{z}-z\partial_{z}^{3}P_{z}. \end{array} \end{array}$$ (23)

We can now insert Eq (19) into these equations. This results in a simple product expression relating $\tilde{\tau }$ to $\tilde{u}$. By comparing the result to Eq (12) we can extract the following Greens function in Fourier space:

$$\begin{array}{c} \begin{array}{cc} & \tilde{G}\left(k_{x},k_{y},z\right)=\\ & \frac{e^{-kz}}{2\mu k^{3}}(\begin{array}{ccc} 2k^{2}-\left(2\nu +kz\right)k_{x}^{2} & -(2\nu +kz)k_{x}k_{y} & (1-2\nu -kz)ikk_{x}\\ -(2\nu +kz)k_{x}k_{y} & 2k^{2}-\left(2\nu +kz\right)k_{y}^{2} & (1-2\nu -kz)ikk_{y}\\ -(1-2\nu +kz)ikk_{x} & -(1-2\nu +kz)ikk_{y} & 2\left(1-\nu \right)k^{2}+k^{3}z \end{array}). \end{array} \end{array}$$ (24)

The analytical form of the GF given here allows us to perform 2.5D FTTC in a very efficient manner. We also checked both by analytically taking the limit of an infinitely thick substrate and by numerical comparison that our method agrees with the one described earlier in the context of a finite thickness elastic halfspace [28].

### Inverse problem and regularization

In principle, a suitable traction field can be found simply by calculating the inverse of $\tilde{G}({\left(k_{mn}\right)}_{x},{\left(k_{mn}\right)}_{y},h)$ and applying the resulting matrix to ${\hat{u}}_{mn}$ to get the traction field ${\hat{\tau }}_{mn}$. However, such a procedure ignores the important fact that the inverse problem of elasticity is ill-posed because elastic effects are long-ranged, meaning that local changes in traction will have non-negligible effects on the displacement even over large distances. This is a problem because the input data for the displacement field will be always subject to experimental noise due to limitations in resolution or inhomogeneities in the medium [4]. A naive inversion will try to reproduce all the fine details of the input field by changing global properties of the force field; this ill-posed nature of the inverse problem will be reflected by a large condition number of the inverse matrix. This problem can be addressed either by filtering the displacement data, e.g. by image smoothing [24, 25], or by introducing a regularization scheme [21].

Because here we focus on the mathematical aspects of TFM, which should be kept separately from issues of image processing, we refrain from smoothing procedures and use a 0^th^ order Tikhonov regularization, which has been found earlier to be very appropriate for the kind of TFM-procedures discussed here [29]. With our regularization approach, Eq (6) leads to

$$\begin{array}{c} \tau \left(x,y\right)=\underset{s}{\text{argmin}}∥\int_{S}G\left(x-x^{\prime },y-y^{\prime },z\right)\cdot s\left(x^{\prime },y^{\prime }\right)\text{d}x^{\prime }\text{d}y^{\prime }-u\left(x,y\right){∥}_{2}^{2}+\lambda^{2}{∥s\left(x,y\right)∥}_{2}^{2}\;. \end{array}$$ (25)

Eq (9) now leads to

$$\begin{array}{c} {\hat{\tau }}_{mn}=[{\left({\tilde{G}}^{†}\tilde{G}+\lambda^{2}I\right)}^{-1}{\tilde{G}}^{†}]{\hat{u}}_{mn}≕{\hat{G}}_{\lambda ,mn}^{\#}{\hat{u}}_{mn}. \end{array}$$ (26)

Here the superscript † designates the Hermitian conjugate. In addition, $\tilde{G}({\left(k_{mn}\right)}_{x},{\left(k_{mn}\right)}_{y},h)$ has been denoted simply by $\tilde{G}$ for visual clarity.

The regularization parameter λ must be chosen with care [23, 30]. If the value is chosen too large, this will result in a loss of accuracy and resolution in the resulting force map. If it is chosen too small, the result will be dominated by noise [22]. Here we use a generalized cross validation technique (GCV) [46] for finding a regularization parameter efficiently. The estimation function is defined by

$$\begin{array}{c} G\left(\lambda \right)=\frac{∥\tilde{G}{\hat{\tau }}_{\lambda }-\hat{u}{∥}_{2}^{2}}{{\left(\text{tr}\left(1-\tilde{G}{\tilde{G}}^{\#}\right)\right)}^{2}} \end{array}$$ (27)

and always has a minimum for a strictly positive value of λ, at which an optimal regularization can be found. It can easily be calculated for a large number of values using the singular value decomposition of $\tilde{G}$ which is known. Using the determined value for λ, an estimate for the deformation field can be calculated.

### Direct method and divergence correction

The direct method for TFM uses the constitutive equation to calculate the local stress tensor of the material from the displacement derivatives or the deformation gradient tensor (compare Fig 1B). The surface traction can be determined from the stress tensor using Eqs (1) and (2). Here we apply this method to the same setup as commonly used with the inverse method. However, the direct method is fundamentally a 3D method and the deformation must be known not only in a single plane, but in a volume below the surface, in order to obtain the full stress tensor. In contrast to the inverse method based on GFs, the direct method can more easily be extended to non-planar surfaces (going beyond Eq (2)) and non-linear material (going beyond (4)).

The strains ∂*u*_*i*_/∂*x*_*j*_ and the components *F*_*ij*_ of the deformation gradient tensor must be obtained numerically. To do so, the displacement field **u** is first sampled on a regularly spaced 3D grid. Then both quantities can be easily obtained using a finite difference scheme. Frank et al [15, 47] determine the derivative by fitting a 1st order polynomial

$$\begin{array}{c} u(x)=ax+by+cz+d \end{array}$$ (28)

to a local region in the resulting traction profile. The components of ***a***, ***b*** and ***c*** contain the strains ∂*u*_*i*_/∂*x*_*j*_ directly. We refer to this technique as the 3x3x3 patch method as a 3 by 3 by 3 data point support is used to estimate the deformation field gradient. We investigate how well this approach performs in comparison to other approximation techniques. The most simple alternative is a simple two-point finite difference method (only the equation for *z*-derivatives is stated):

$$\begin{array}{c} \begin{array}{l} \frac{\partial u_{i}}{\partial z}\begin{array}{c} {|}_{klm}\approx \frac{u_{i,kl\left(m+1\right)}-u_{i,kl\left(m-1\right)}}{2\Delta x}. \end{array} \end{array} \end{array}$$ (29)

Increasing the number of sampling points contributing to the derivatives as done in Eq (28) should in theory decrease the uncertainty of the result, but will decrease resolution. Another alternative to this constitutes a four-point scheme:

$$\begin{array}{c} \frac{\partial u_{i}}{\partial z}{|}_{klm}\approx \frac{-u_{i,kl(m+2)}+8u_{i,kl(m+1)}-8u_{i,kl(m-1)}+u_{i,kl(m-2)}}{12\Delta x}. \end{array}$$ (30)

In all three cases special non-symmetric expressions are used close to the boundary that make use of the same number of support points, but avoid contributions outside the observed area. All methods for numerical derivatives are dependent on the sampling distance defined by the sampling lattice. A smaller sampling distance should reduce the systematic error, but on the other hand the statistical error on the deformation gradient is proportional to the ratio between the statistical error of the displacement field and the spacing and therefore will increase for smaller sampling distance.

While the inverse method always gives a valid displacement field because it is calculated from a force distribution as a direct problem, the direct method calculates a displacement field that might not be valid as it violates force and torque balances. In a static system, the force balance leads to the Cauchy momentum equation [45] (here stated in the form used in linear elasticity):

$$\begin{array}{c} \frac{\partial \sigma_{ij}}{\partial x_{j}}=0\;. \end{array}$$ (31)

In other words the stress tensor must be divergence-free (*solenoid*). This property is not restricted to a specific geometry of the system. It describes a fundamental property resulting from the fact that the stress tensor represents local force densities that must be in balance for a static situation free of external forces. The coordinate of the initial deformation fields where the corresponding stress tensor field satisfies Eq (31) are called *compatible* [45]. The importance of this condition has been pointed out before in the context of monolayer stress microscopy [48] and 3D TFM [17].

While the physical deformation field **u**^(*T*)^ is always compatible, the measured deformation field **u**^(*C*)^ is not, as it contains errors attributed to noise and thus the obtained stress tensor may not satisfy Eq (31). If we describe *σ*_*ij*_(**u**) to be the stress tensor obtained from a deformation field **u**, we easily see by

$$\begin{array}{c} \frac{\partial \sigma_{ij}\left(u^{\left(C\right)}\right)}{\partial x_{j}}\frac{=\partial \left(\sigma_{ij}\left(u^{\left(C\right)}\right)-\sigma_{ij}\left(u^{\left(T\right)}\right)\right)}{\partial x_{j}}+\underset{=0}{\underset{︸}{\frac{\partial \sigma_{ij}\left(u^{\left(T\right)}\right)}{\partial x_{j}}}} \end{array}$$ (32)

that only the noise part of the deformation field will contribute to the divergence of the obtained stress tensor *σ*_*ij*_(**u**^(*C*)^). This means that we can use this divergence to determine what ratio of the input data is attributed to noise. The divergence of the calculated stress tensor *σ*_*ij*_(**u**^(*C*)^) can be calculated on a local basis by using a symmetric two-point form.

Instead of the tensor based formulation Eq (31), the compatibility equation can also be represented as a condition for each column of the stress tensor:

$$\begin{array}{ccc} \nabla \cdot a=0, & \nabla \cdot b=0, & \nabla \cdot c=0, \end{array}$$ (33)

where we defined $a={\hat{e}}_{1}\sigma$, $b={\hat{e}}_{2}\sigma$ and $c={\hat{e}}_{3}\sigma$. This observation immediately generates a relation to hydrodynamics, because a similar situation can be found for hydrodynamic descriptions of incompressible fluids, where the mass conservation equation reduces to

$$\begin{array}{c} \nabla \cdot v=0 \end{array}$$ (34)

for a flow field **v**. This property has been exploited by various studies and using a variety of techniques to remove potential noise from ***v*** [49–51]. Because of the relation between the stress tensor and the surface traction Eq (1), removing the noise contributions from *σ*_*ij*_ for the full substrate might also yield a better result for the surface traction, similar to regularization for the inverse method. An efficient method for performing this kind of divergence correction on a flow profile has been described by Wang et al. [52] and this method can be generalized to any divergence-free vector field. A short description of their technique is given in the S1 Appendix. This technique makes use of the particular structure of the problem to result in an algorithm that applies matrix operations only on one coordinate at the same time, which strongly reduces computational complexity as well as avoids complex operations like matrix inversion.

The noise removal technique now consists of an iterative process. We start with the initial condition ***σ***^(0)^ ≔ ***σ***_*exp*_ and *i* ≔ 0 and iterate the following steps:

1. Split the stress tensor into column vectors: (**a**_*exp*_, **b**_*exp*_, **c**_*exp*_) = ***σ***^(*i*−1)^
2. Find **a**_*c*_, **b**_*c*_ and **c**_*c*_ that satisfy Eq (33) using the mentioned procedure for vector fields using **a**_*exp*_, **b**_*exp*_ and **c**_*exp*_ as input.
3. Reassemble the vector fields into a new tensor **w** = **w**^(*i*)^ ≔ (**a**_*exp*_, **b**_*exp*_, **c**_*exp*_).
4. Obtain a symmetric approximation for the stress tensor ***σ***^(*i*)^ = 1/2(**w** + **w**^*T*^).

In each iteration, the divergence of the resulting stress tensor ***σ***^(*i*)^ is reduced. We tested this reduction for a number of different inputs, including a white noise input, and found that a fixed iteration of 20 cycles was sufficient to remove the noise of the input (compare S1 Appendix).

## Results

### Design of simulated traction patterns

**Fig 2:**
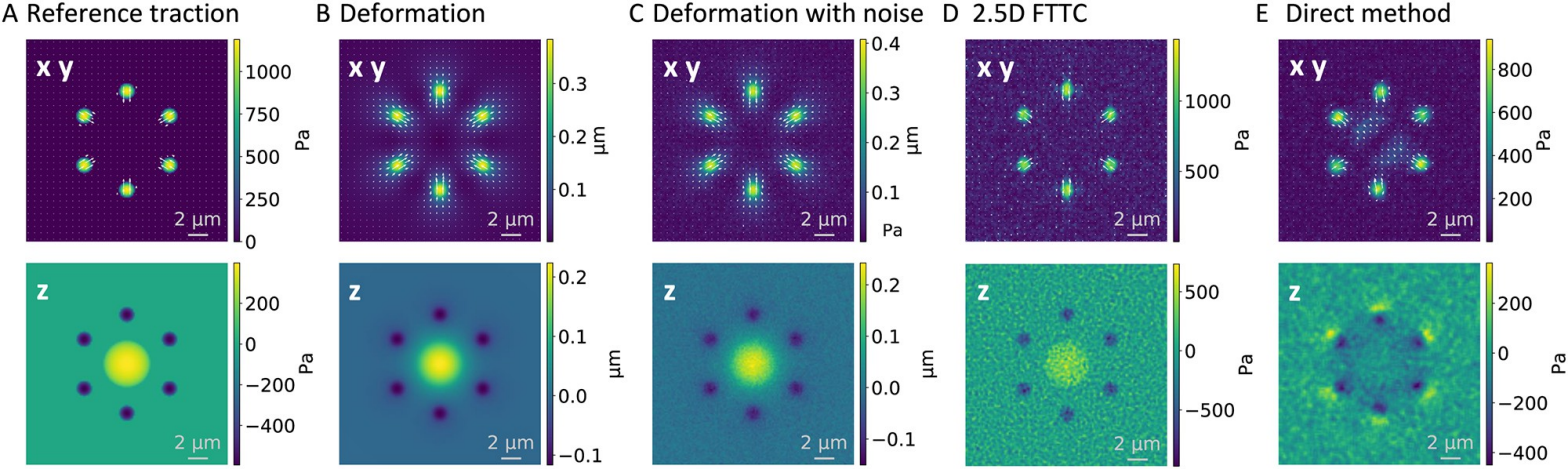
Workflow for reconstruction for cell-like traction pattern. (A) As an introductory example, we include the two most important features of adherent cells, namely focal adhesions with mainly tangential traction and the normal push of the nucleus into the substrate. The plots in the upper row show the tangential components, while the lower row contains the normal component. (B) From this given traction field one can then calculate the analytical solution for the deformation field. (C) Noise is added to simulate experimental data (here ***σ***_*N*_/< ∥*u*∥ > = 0.2). (D) For this low noise level, 2.5D FTTC works very well. (E) The direct method gives similar results for the tangential tractions, but performs less well for normal traction. Details on the simulation parameters for this profile can be found in the S1 Appendix.

Because here we aim at testing different traction force reconstruction methods, we design traction patterns that are useful for the task at hand and for which we can calculate the deformations analytically. We then add displacement noise to these solutions and finally reconstruct the traction and compare with the original pattern. This process is illustrated in Fig 2 for an example that includes the two most important features known from adherent cells, namely tangential traction at focal adhesions and the normal push by the nucleus (which due to momentum conservation has to be balanced by counteracting normal forces at the focal adhesions). We discuss the properties of the different reconstructions in the subsequent section. For the analytically tractable patterns, we choose linear combinations of Hertz-like patches:

$$\begin{array}{c} \tau_{\text{Patch}}=\frac{3}{2\pi a^{3}}Q\sqrt{a^{2}-{\left(x-x_{0}\right)}^{2}-{\left(y-y_{0}\right)}^{2}}\Theta \left(a^{2}-{\left(x-x_{0}\right)}^{2}-{\left(y-y_{0}\right)}^{2}\right), \end{array}$$ (35)

where the force transmitted by the patches is represented by **Q**, the adhesion size *a* and the positions *x*_0_ and *y*_0_ are parameters that can be chosen freely, and Θ is the Heaviside function. We call these traction profiles *Hertz-like* because they match the profiles in the Hertz and Cerrutti contact problems. The traction fields for this problem have been extensively studied for materials that obey linear elasticity [53]. Based on an approach suggested by [54], we derive the full 3D solution for Eq (35) in the S1 Appendix.

The analytical solution is then sampled on an lattice grid with cuboid unit cells, where the distance between sampling points in *x* and *y* direction is equal. The distance in z-direction is usually chosen larger to reflect the anisotropy of the point spread function of traditional optical microscopes. In the next step, Gaussian noise is added to the displacements. For each data point in the grid sampled deformation field a random number is added. This number is drawn from a Gaussian distribution with mean *μ* = 0 and standard deviation *σ* being fixed for all sampling points and chosen with respect to the amplitude of deformation averaged over the whole field, which we designate as < ∥*u*∥ >. In the following, we give the magnitude of the noise in *σ*/< ∥*u*∥ >. It has been show before that the noise distribution does indeed have a Gaussian shape [55]. The perturbed fields now form the input for the actual TFM-analysis. The determined traction profile ***τ***^*recon*^ can then be compared to the initial analytical profile ***τ***. Studies that describe new methods to improve the image processing part of TFM often include a simulation of the bead distribution, sometimes also assuming a specific point spread function [18, 56, 57]. However, noise can also arise from different sources, e.g. inhomogeneities in the gel or the bead distributions. Because here we do not aim at simulating experimental setups, but focus on the mathematical properties of different TFM-procedures, we simply simulate displacement noise [21, 23, 29]. Every TFM-method will eventually fail at very high noise levels, but here we ask if direct or inverse methods perform better for low or high noise levels. For each dataset used, we list the full set of parameters, including the sample point spacing, the material parameters and the parameters of the Hertz-like patches superposition to generate the profile in the S1 Appendix.

### Performance of direct method

**Fig 3:**
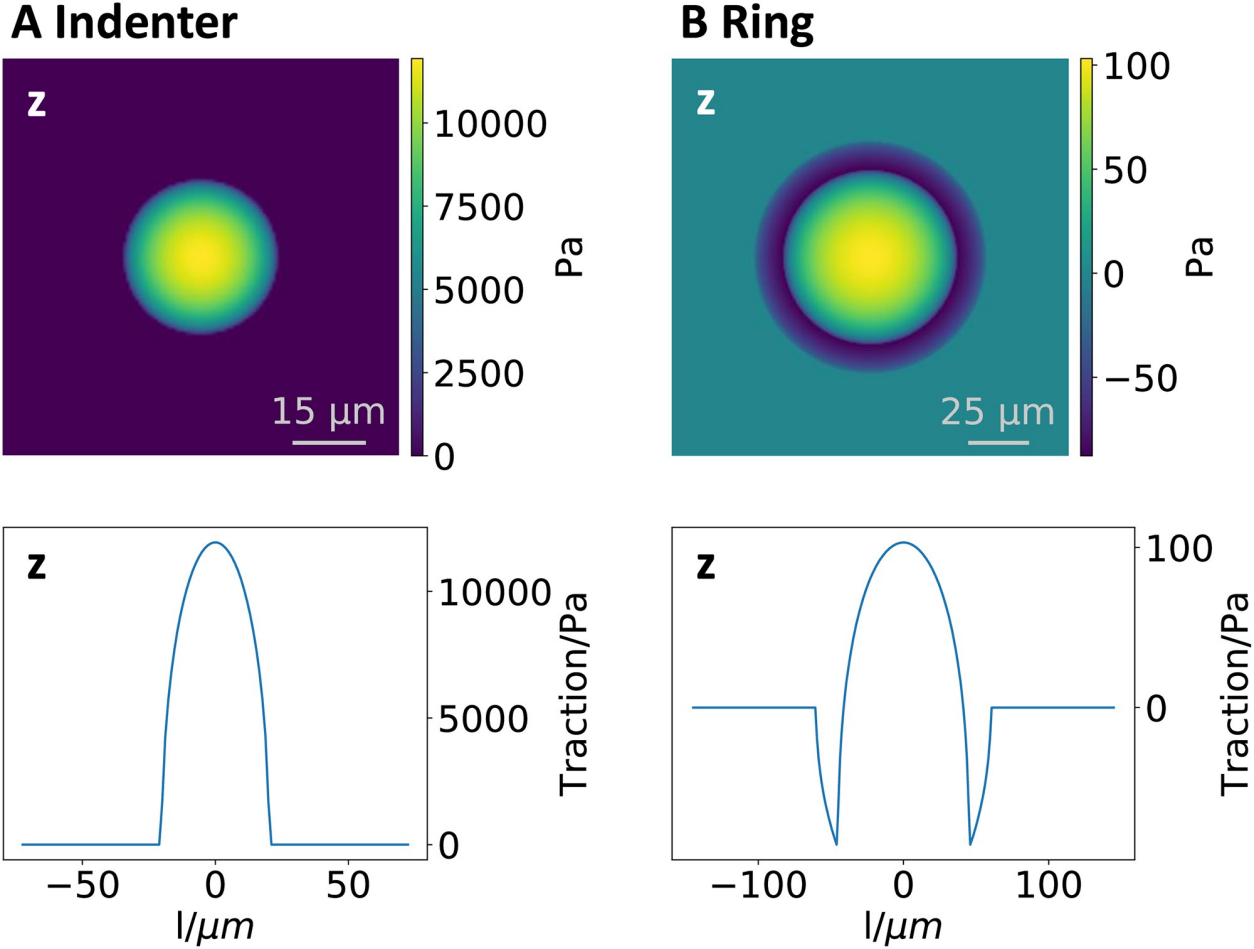
Two different Hertzian traction profiles used for normal analysis with the direct method. (A) Indenter monopole with *F*_*z*_ = 10Â *μN*. (B) Ring dipole with *F*_*z*_ = 0 *μN*. The upper row present a heat map of the normal traction *τ*_*z*_ at the substrate surface. There is no tangential traction in these cases. The lower row present the change of the normal traction along a lower-left to upper-right diagonal section. The results of the analysis corresponding to these profiles are presented in Fig 4. The other simulation parameters for these profiles can be found in the S1 Appendix.

We first optimize the direct method by assessing the performances of the numerical derivatives and the importance of a divergence correction. Towards this aim, we choose traction profiles that emphasize the normal component. This is not the standard case in TFM, but in this way, we can best test the performance of the different approximations for derivatives that are required for the direct method. Moreover, this choice demonstrates our ability to deal with three dimensions. Fig 3 shows the two simple Hertzian traction patterns investigated below together with the cell-like profile presented in Fig 2. The first profile corresponds to a simple Hertz contact, which means that it is a force monopole pushing into the substrate. This setup could be recreated experimentally using a spherical indenter that presses into the substrate. Note that in contrast to FTTC, the direct method works in real space and also can reconstruct force monopoles. The second profile mimics a situation in which pushing and pulling tractions are exerted in different regions in a ring-like pattern. For example, this resembles the way cancer cells invade tissue with invadopodia or funghi invade plants.

To quantitatively compare the different methods of determining the deformation gradient as well as the effects of our divergence correction scheme, we calculate different quantities of interest. First we calculate the force monopole components *F*_*x*_, *F*_*y*_, *F*_*z*_ defined by

$$\begin{array}{c} F_{i}=\int_{S}\tau_{i}\left(x,y\right)\text{d}x\;\text{d}y \end{array}$$ (36)

which describe the total force transmitted between the sample and the substrate in our field of view. Numerically, the integration is performed using the Simpson formula [58]. These quantities serve as indicators on numerical inaccuracies that add up. If they differ from their predicted value, they will therefore indicate asymmetric and systematic errors in the analysis. The amplitude of the tangential components *F*_*x*_ and *F*_*y*_ should be zero for all our normal indentation profiles, as no tangential force is transmitted. Due to symmetry, we expect the *F*_*y*_ component to show the same behavior as the *F*_*x*_ component. The *F*_*z*_ component should vanish for the dipolar patterns. If *F*_*z*_ was different from the expected value, this may indicate a systematic error due to the non-symmetric way of taking the derivative at the surface.

Next we calculate the total L2-difference between the TFM results and the reference defined as

$$\begin{array}{c} d_{L2}=∥\tau^{recon}-\tau^{true}{∥}_{2}=\sqrt{\int_{S}|\tau^{recon}\left(x,y\right)-\tau^{true}\left(x,y\right){|}^{2}\text{d}x\;\text{d}y}. \end{array}$$ (37)

This quantity measures how well the reconstructed field matches the analytical solution. Again, the integration is performed using the Simpson formula. In contrast to the monopole, this value will not only capture systematic aberration offset, but also general noise and gives a measure for the uncertainty of the results. A high *d*_*L*2_ indicates that the reconstruction contains a high amount of noise artifacts.

**Fig 4:**
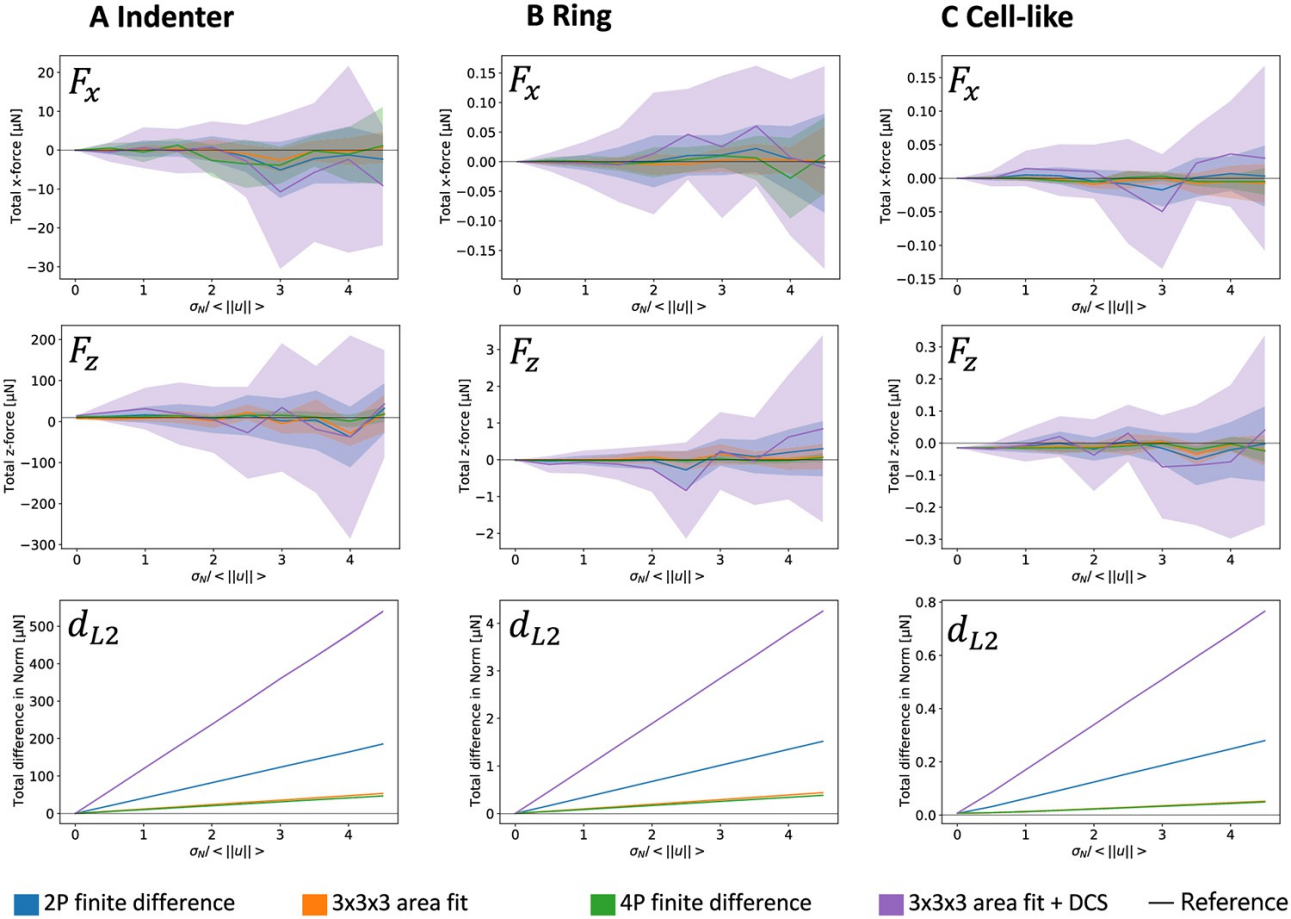
Quantitative comparison between different variants of the direct method. The plots use the Hertzian traction profiles introduced in Figs 2 and 3. A normalization factor < ∥*u*∥ > used to compare noise levels for different amplitudes is calculated by taking the mean of the amplitudes of the deformation field. In the upper line, we plot the variation in the predicted total force in *x* direction as a function of the standard deviation of Gaussian noise added to the input data. The line indicates the mean and the colored area indicates the standard deviation. In the mid row the same is done for the total traction in *z*-direction. In the lower row, we plot the total difference in norm, where the variation between the different samples is shown to be negligible. The different colors designate the different ways of calculating the deformation gradients as well as whether the divergence correction scheme (DCS) is used. *F*_*y*_ is not shown due to its similarity to *F*_*x*_.

Fig 4 shows the performance of the different variants of the direct method as assessed through these metrics. First we observe that as expected, the variance in the monopoles and the distance metrics increase with increasing noise levels such that *σ*_*N*_/< ∥*u*∥ > = 1 has to be considered to be large noise. At much higher noise levels, all methods will fail. For the DM investigated here, we find that using a four-point (4P) form instead of a simple two-point (2P) form does not improve the result, but causes a significantly higher level of overall noise, likely due to overfitting. In contrast, using the 3x3x3 patch fit significantly improves the noise suppression, both in the full field as well as the background. However, it will result in an underestimate for the force monopole z-component *F*_*z*_ in case of the indenter profile (A). This is likely due to the fact that *u*_*z*_/*z* will be underestimated due to the non-symmetric derivative. Applying the divergence correction algorithm does improve the result in this case and also offers a slightly better noise reconstruction. However, an opposite effect is found for *F*_*z*_ for profiles (B) and (C), where the noise removal introduces a systematic offset in the normal traction component, as well as for *F*_*N*_, where it also increases the aberration in the force monopole normal components. Notably the difference in norm describes a straight line with negligible variance between the samples. This comes from the fact that for all methods, the deformation gradient and therefore also the resulting stress have a linear relation to the input deformation field. This implies a linear relation in the variance due to noise. This proportionality is then shared by the *d*_*L*2_ parameter. Although the divergence correction ensures that formally force and torque balance are satisfied, it does not improve the performance of the DM.

### Comparison of direct method and FTTC

Now that we have optimized the direct method, we next compare it to the inverse method, namely with FTTC-calculations both in 2.5D (2.5D FTTC) as well as with calculations in which contributions in the normal dimension are not considered, as described by Eq (10) (2D FTTC). Because we now compare with FTTC, we work with the standard choice for TFM on planar substrates, namely a collection of circular adhesion sites with mainly tangential tractions, as commonly observed for contractile cell types which adhere to flat substrates through focal adhesions.

**Fig 5:**
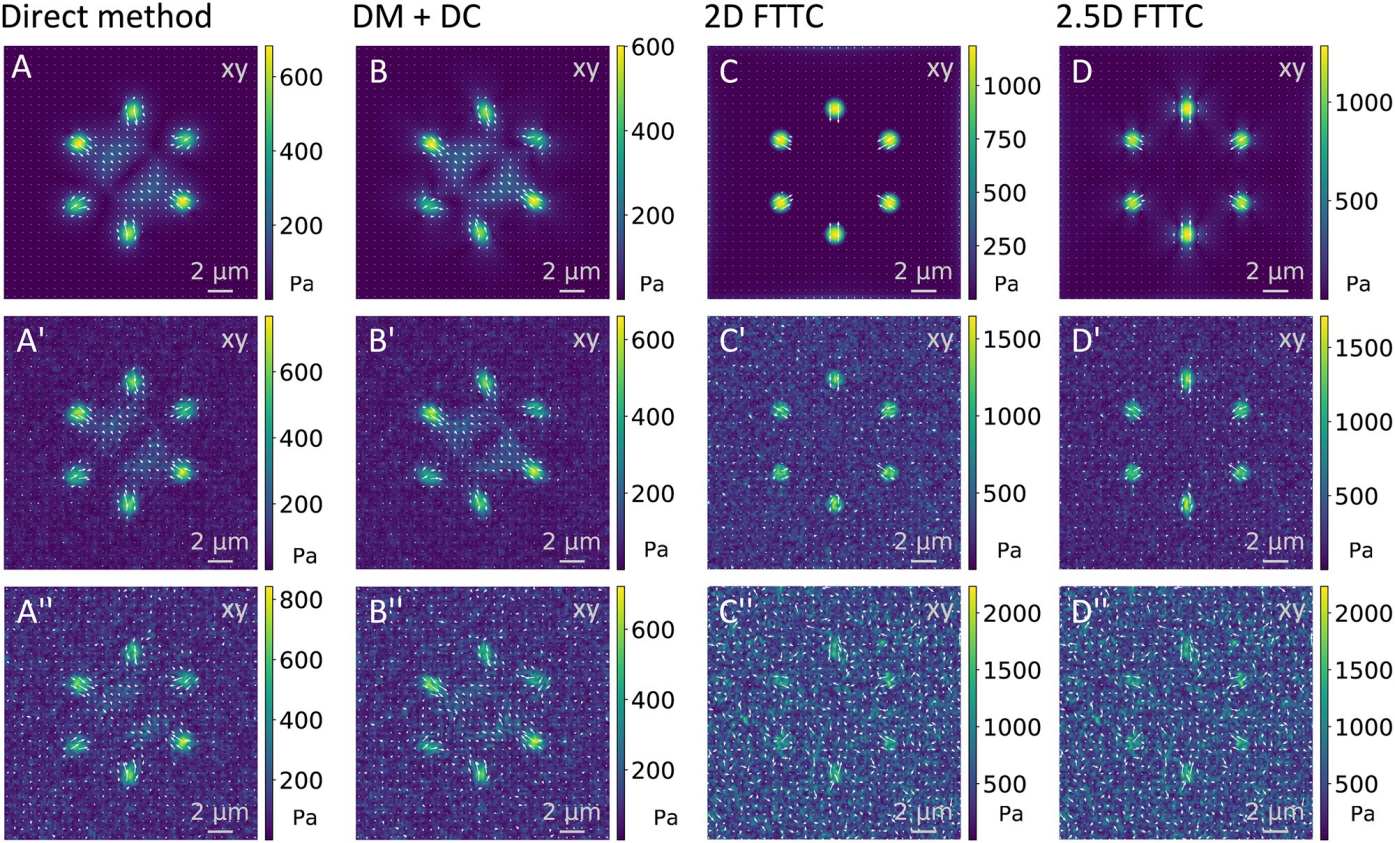
Reconstruction for cell-like traction pattern for different noise levels and reconstruction methods. The images show the reconstruction for no (upper row), large (*σ*_*N*_/< ∥*u*∥ > = 1, mid row) and very large (*σ*_*N*_/< ∥*u*∥ > = 2, lower row) Gaussian noise added to the analytical solution for the displacement before reconstruction with the different methods as indicated. The simulation parameters are identical to the ones used in Fig 2 and details can be found in the S1 Appendix.

While Fig 2 showed traction reconstructions with different methods for the cell-like pattern for small noise (*σ*_*N*_/< ∥*u*∥ > = 0.2), in Fig 5 we now show such reconstructions for variable noise levels (*σ*_*N*_/< ∥*u*∥ > = 0, 1 and 2 from top to bottom). For simplicity, here we only show the components in the xy-plane. A visual inspection suggests that FTTC reconstructs the shape of the deformation profile most faithfully for low noise levels. For high noise levels, it predicts a too high level of force. For very high noise levels, the adhesion sites can be more easily identified by the DM compared to FTTC. The DM and in particular the DM using divergence correction seem to predict a traction strength amplitude that is largely independent of noise. On the other hand, the divergence correction scheme produces some artifacts, independent of the noise level. This suggests that the divergence removal algorithm converts local noise into a more distributed signal that is not directly related to the physical force generators.

**Fig 6:**
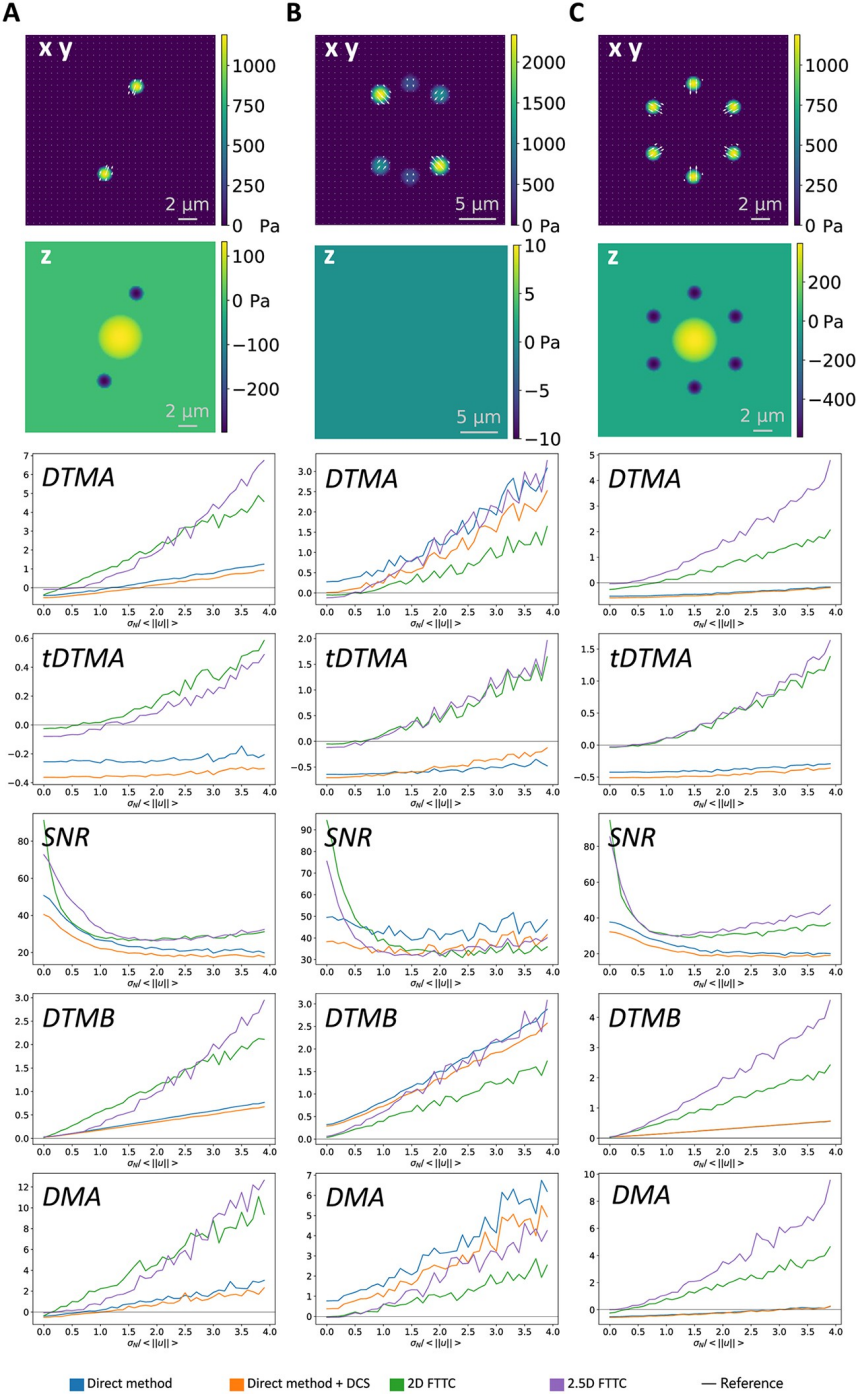
Quantitative comparison of direct and inverse methods. Performance of different force reconstruction techniques as a function of noise for different force profiles. The point profiles tested are shown in the top two row: In the first row, the heat map indicates the amplitude of the the tangential traction, while the white arrows indicate their direction. In the second row, the heat map indicates the normal traction. Below are different metrics shown, describing the quality of the reconstruction at different locations. The plots in one column all correspond to the same force profile. Details on the simulation parameters for all three profiles can be found in the S1 Appendix.

In order to make these qualitative assessments more objective, we next evaluated a series of established metrics for the three different traction profiles shown in the upper two rows of Fig 6. As before, the analytical solutions are known and it is easy to add Gaussian noise to the resulting deformation fields. The analysis results in a traction field described by a discrete sample vector $\tau_{j}^{\text{recon}}$ for each site sampling point *j*, that can be compared to its theoretical equivalent $\tau_{j}^{\text{true}}$ predicted from the analytical solution. We estimate the accuracy of the different reconstructions using the following five metrics [23, 29]:

- The deviation of traction magnitude at adhesions (DTMA) is given by:
  $$\begin{array}{c} \text{DTMA}=\frac{1}{N_{P}}\sum_{i}\frac{{\text{mean}}_{j(i)}(∥\tau_{j(i)}^{\text{recon}}{∥}_{2}-∥\tau_{j(i)}^{\text{true}}{∥}_{2})}{{\text{mean}}_{j(i)}(∥\tau_{j(i)}^{\text{true}}{∥}_{2})}. \end{array}$$ (38)
  Here *N*_*p*_ is the number of adhesion patches and the index *i* iterates over the individual patches. For each patch, the mean is taken over all sampling points *j*(*i*) belonging to the given patch. The DTMA determines how well the average magnitude of the patches is predicted. A good reconstruction would yield an DTMA close to zero, while a positive or negative value would indicate an over- or underestimation, respectively.
- The deviation of traction magnitude at adhesions restricted to the two tangential dimensions (tDTMA) is given by:
  $$\begin{array}{c} \text{tDTMA}=\frac{1}{N_{P}}\sum_{i}\frac{{\text{mean}}_{j(i)}(n_{2}\left(\tau_{j(i)}^{\text{recon}}\right)-n_{2}\left(\tau_{j(i)}^{\text{true}}\right))}{{\text{mean}}_{j(i)}(n_{2}\left(\tau_{j(i)}^{\text{true}}\right))}. \end{array}$$ (39)
  Here *N*_*p*_ is the number of adhesion patches and the index *i* iterates over the individual patches and $n_{2}\left(\tau \right)=\sqrt{\tau_{x}^{2}+\tau_{y}^{2}}$. For each patch, the mean is taken over all sampling points *j*(*i*) belonging to the given patch. The tDTMA determines how well the average magnitude of the patches is predicted. In contrast to DTMA, we do only take into account the two tangential components. This focuses on situations in which the effect on the normal component behaves differently from the tangential one. A good reconstruction would yield an tDTMA close to zero, while a positive or negative value would indicate an over- or underestimation, respectively.
- The signal to noise ratio (SNR) is defined by:
  $$\begin{array}{c} \text{SNR}=\frac{\frac{1}{N_{p}}\sum_{i}{\text{mean}}_{j(i)}(∥\tau_{j}^{\text{recon}}{∥}_{2})}{{\text{std}}_{k}(∥\tau_{k}^{\text{recon}}{∥}_{2})}. \end{array}$$ (40)
  Here *i*, *j*(*i*) and *k* are defined as above. The signal to noise ratio describes how well the adhesion sites are realized in comparison to background noise. The value should be significantly larger them 1 to indicate a good separation between traction sites and noise.
- The deviation of traction magnitude in the background (DTMB) is given by:
  $$\begin{array}{c} \text{DTMB}=\frac{{\text{mean}}_{k}(∥\tau_{k}^{\text{recon}}{∥}_{2})}{\frac{1}{N_{p}}\sum_{i}{\text{mean}}_{j(i)}(∥\tau_{j}^{\text{true}}{∥}_{2})}. \end{array}$$ (41)
  Here *k* runs over all sampling points not belonging to any patch. *i* and *j*(*i*) again iterate over the patches and their sampling points respectively. The DTMB describes the level of background noise in the reconstruction. Ideally it takes a value close to zero indicating a low level of artifacts in the background.
- The deviation of traction maximum at adhesions (DMA) is defined by:
  $$\begin{array}{c} \text{DMA}=\frac{1}{N_{P}}\sum_{i}\frac{{\text{max}}_{j(i)}(∥\tau_{j(i)}^{\text{recon}}{∥}_{2})-{\text{max}}_{j(i)}(∥\tau_{j(i)}^{\text{true}}{∥}_{2})}{{\text{max}}_{j(i)}(∥\tau_{j(i)}^{\text{recon}}{∥}_{2})}. \end{array}$$ (42)
  Again *i* and *j*(*i*) are defined as above. The DMA is similar to the DTMA, but rather them using the average traction over the whole adhesion site, the peak traction is taken into account. This emphasizes the reconstruction of the correct amplitude in core area over the correct profile close to the boundary. Like with the DTMA a good reconstruction would yield a DMA close to 0, while a positive or negative value would indicate an over- or underestimation, respectively.

Fig 6 shows the performance of the three different methods as a function of increasing noise level and for different traction patterns as shown in the top row. For all metrics except the SNR, the optimal value is shown as black horizontal line. In general, we see that all methods fail at very high noise levels. We find that FTTC is better than the DM in predicting the correct strength of the adhesions at low noise levels as seen in the DTMA, tDTMA and DMA results. However, the situation is reversed for higher noise, as the regularization is not sufficent to prevent overfitting above a certain noise level. While the application of the divergence correction scheme does reduce the SNR result, it improves the results for DTMB and DMA, by bringing them closer to zero, particular for high noise levels. A beneficial effect of the divergence removal is to reduce the difference between the DTMA and tDTMA scores, meaning that the algorithm does predict the orientation of the force vector more correctly. In contrast, the FTTC algorithm shows significantly better values for the DTMB metric, which means that it is more effective in preventing artifacts in the outside of adhesion sites. Surprisingly, the signal to noise ratio is lower in the case of FTTC compared to the direct method. This can be attributed to the fact that FTTC works in Fourier space and errors in the reconstruction effect the whole field of view, not only the area close to the adhesion sites. We conclude that both methods perform similarly well, that the divergence correction as used here is in fact a disadvantage, that FTTC works best for small noise and that for larger noise the direct method becomes comparable and gives a clearer visualisation due to the higher SNR.

### Effect of sampling density

**Fig 7:**
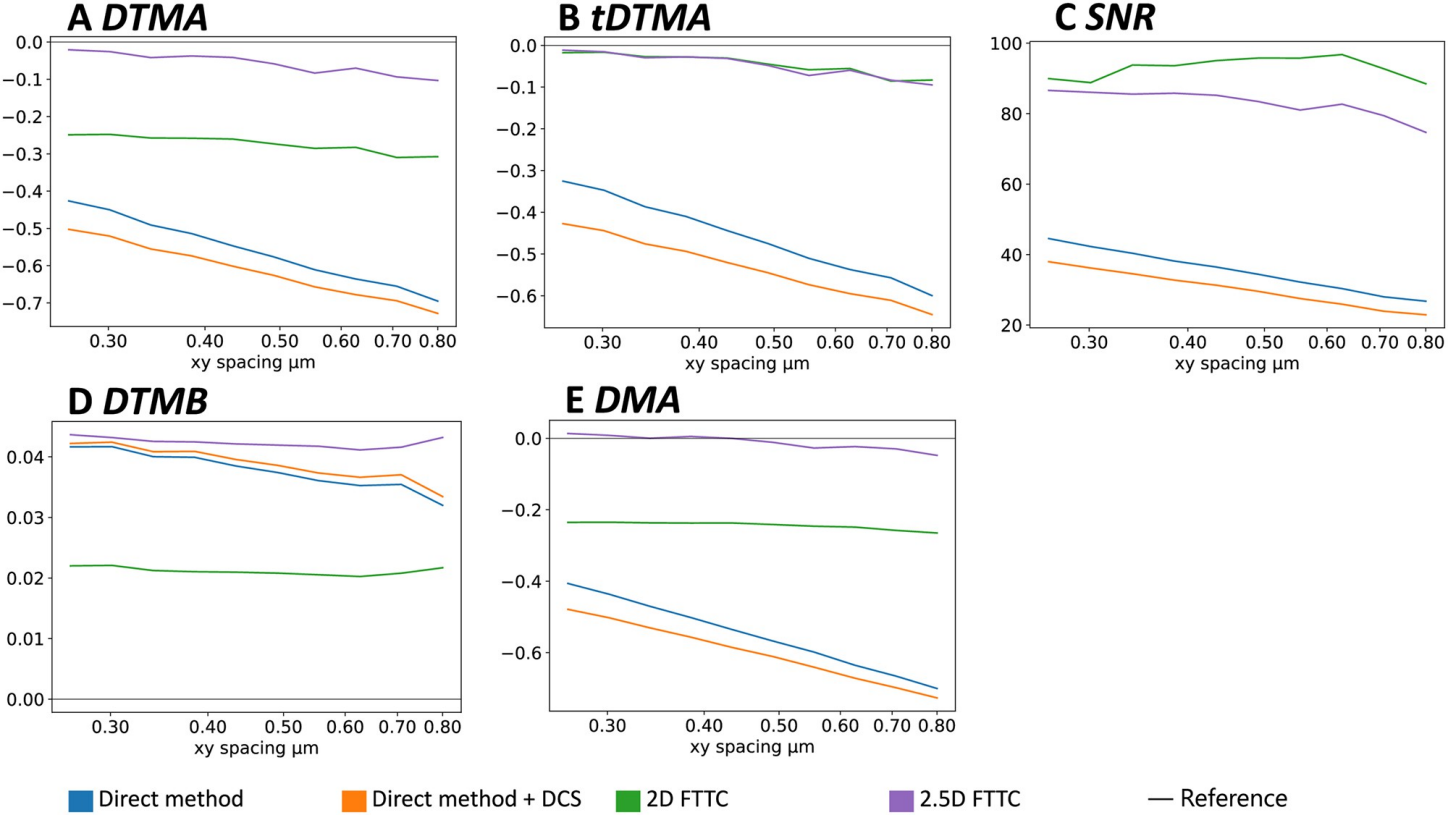
Effects of variation in sample density. Plots A to E show how the different metrics are affected when using a different distance of the sampling points when setting up the input data without adding any noise. The profile first shown in Fig 2 is used. The default sample point spacing in *x* and *y* for this profile is 0.4 μm and here is varied up and down. The z-component is scaled proportionally. Since there is no noise, in general FTTC performs much better. The direct method shows a consistent improvement for decreasing sampling point distance. Details on the simulation parameters can be found in the S1 Appendix.

We finally study the effect of variation in sampling density, which experimentally is related to marker bead density and the resolution of the optical microscope. Because in practise displacement noise is expected to change with sampling distance and because we now focus on the effect of sampling density, in Fig 7 we show the results for vanishing displacement noise. As expected, overall all metrics become worse with increasing sampling distance. Interestingly, however, the performance of FTTC seems to be more robust, while the DM quickly decreases in performance. This implies that a decrease in sampling distance (that is an increase in sampling density) will be much more beneficial for the DM. We note that for FTTC, the SNR and (for 2.5D FTTC) also DTMB can even slightly improve with decreasing sampling density. This surprising (but weak) effect might be related to the fact that for the Fourier method, increasing sampling distance amounts to stronger filtering of the data, thus focusing on the overall adhesion pattern. We also checked that the same trends persist for variation of sampling distance at finite noise (S1 Appendix). We noticed that for FTTC the SNR now significantly improves for a decrease in sampling density. This confirms that noise and sample point density are in fact correlated, because an increase in sampling density also increases the number of nodes that contribute noise towards the calculation. FTTC and DM are affected differently by an increase in sampling density in the presents of noise. For the DM the increase of the numbers of nodes improves the accuracy of the numerical gradients. For FTTC, the quality of reconstruction at the sampling points does not significantly improve when increasing the resolution, but the increase of the number of nodes increases the negative effect of noise on the result.

## Discussion

Motivated by the observation that different TFM-methods are often advanced in specific contexts, but rarely compared to each other, here we have conducted an in-depth comparison of inverse and direct methods in the framework of 2.5D TFM. Recently such a comparison has been conducted using FEM-approaches for 3D TFM [17, 18], but here we focus on 2.5D TFM as the traditional setup for high-resolution experiments. For the inverse method, we have used the commonly used method, namely the Fourier method FTTC, which is very fast and reliable when combined with a regularization scheme, for which here we have chosen zero-order Tikhonov regularization with generalized cross-validation for identification of the regularization parameter λ. Although we here work with the setup of a flat elastic substrate, which usually is treated with 2D TFM, in order to account for the 3D-nature of the direct method, we included the third dimension by considering also normal forces (2.5D TFM). For this purpose, we have implemented a version of 2.5D FTTC based on newly derived GFs with normal components. With these advances for 2.5D FTTC, the two methods can be directly compared to each other.

For each performance test, we have first designed traction patterns that are representative for experiments, suitable for the task at hand and analytically tractable. Motivated by the observation that experimental noise is Gaussian-distributed in experiments [55], we have added Gaussian noise to the displacements and then performed the reconstructions with different methods. In the future, this procedure could be complemented by a stronger focus on the actual image generation occurring in TFM-experiments, in particular by using specific image processing algorithms and point spread functions [18, 56, 57].

We have evaluated our reconstructions using commonly used metrics [23, 29]. This methodology then was used in three ways. We first optimized the direct method, then compared it with FTTC, and finally studied the effect of sampling distance. For the direct method, we found that the 3x3x3 patch calculation of the derivatives is indeed the best solution and that the standard divergence correction from hydrodynamics works, but does not necessarily improve our solutions and in fact worsens the visual appearance of the traction pattern. Assuming a perfect elastic material, divergence is generated only by noise, which is known to be essentially Gaussian in experimental TFM data. This means that it is uncorrelated between different dimensions. Divergence removal however couples the different dimensions and therefore does not counteract the process that generated the divergence. We conclude that although required from the viewpoint of elasticity theory, divergence removal is not really needed for the TFM procedures used here.

Our main result is the demonstration that the direct method for TFM can be used to reliably predict the traction field at the surface of a flat elastic substrates and in fact performs comparatively well to the best inverse method, that is FTTC, at least for large noise, when FTTC worsens more quickly. The direct method also offers an interesting alternative to costly FEM-simulations, in situations where FTTC cannot be applied. The 3x3x3 patch method that has proven to be a reliable method for deformation gradient calculation can in fact be easily adapted for curved surfaces. Although FTTC is expected to remain the standard method for 2D TFM, we believe that the direct method is a valuable alternative even in this case, e.g. because it can also reconstruct monopolar traction patterns. For 2.5D TFM we believe that the choice between the direct and inverse methods should depend on context, but that in principle, both might work well.

In the future, the direct method might become more important due to several experimental developments. Recent advances in microfabrication and additive manufactoring will lead to completely new geometries and setups [32–35], for which the analytical solutions required for methods like FTTC will not be possible anymore, except for simple geometries like elastic beads [12, 36]. Inverse methods implemented in FEM-environments are the method of choice in this case, but because they are computational costly, the DM is a attractive alternative. Moreover image quality for TFM quickly improves, e.g. due to super-resolution microscopy [26, 37–40], and this might play in favor of the direct method, which without displacement noise becomes better with smaller sampling distance.

## Supporting information

S1 Appendix Supplementary calculations related to the Hertz-like force profile, details of the divergence correction algorithm and parameters used for the simulated profiles. (PDF)
