# Supplementary material for: Comparison of direct and inverse methods for 2.5D traction force microscopy
Source: PLoS One. 2022 Jan 20;17(1):e0262773. doi: 10.1371/journal.pone.0262773 (PMC8775276; doi:10.1371/journal.pone.0262773)
Supplement: S1 Appendix — (PDF) [file pone.0262773.s001.pdf]

Comparison of direct and inverse methods for 2.5D traction force microscopy – Supporting information –

Johannes W. Blumberg and Ulrich S. Schwarz

S1 Divergence correction of a vector field

To find a divergence correct vector field, the input vector field $\mathbf{u}_{exp}$ is silty perturbed into a corrected vector profile $\mathbf{u}_c$, which is free of divergence. A corrected profile can therefore be obtained by solving the optimization problem

$$\mathbf{u}_c = \underset{\mathbf{u}}{\operatorname{argmin}} \int (\mathbf{u}_{exp}(\mathbf{x}) - \mathbf{u}(\mathbf{x}))^2 \, \mathrm{d}^3 x \tag{S1}$$

under the constraint

$$\nabla \cdot \mathbf{u}_c = 0. \tag{S2}$$

Discretized to a mesh using a first order finite difference, the problem can be written [51]:

$$(u_c^{ijk}, v_c^{ijk}, w_c^{ijk}) = \underset{(u,v,w)}{\operatorname{argmin}} \sum_{i,j,k=1}^{n_x,n_y,n_z} (u_{exp}^{ijk} - u^{ijk})^2 + (v_{exp}^{ijk} - v^{ijk})^2 + (w_{exp}^{ijk} - w^{ijk})^2 \tag{S3}$$

under the constraint

$$\sum_{i'=1}^{n_x} d_{n_x}^{ii'} u_c^{i'jk} + \sum_{j'=1}^{n_y} d_{n_y}^{jj'} v_c^{ij'k} + \sum_{k'=1}^{n_z} d_{n_z}^{kk'} w_c^{ijk'} = 0 \quad \text{for all } i,j,k = 1 \ldots n_x, n_y, n_z. \tag{S4}$$

Here u_c^{ijk},v_c^{ijk} and w_c^{ijk} denote the three vector components sampled on a grid and $d_{n_x}^{ii'}$,$d_{n_y}^{jj'}$ and $d_{n_z}^{kk'}$ are the coefficients of the one-dimensional finite difference operators in x, y and z direction. The coefficients $d_{n_x}^{ii'}$s form $n_x \times n_x$ sparsely populated matrices $\mathbf{d}_{n_x}$ given by

$$\mathbf{d}_{n_x} = \frac{1}{\Delta x} \begin{pmatrix} -1 & 1 & & & \\ -\frac{1}{2} & 0 & \frac{1}{2} & & \\ & \ddots & \ddots & \ddots & \\ & & -\frac{1}{2} & 0 & \frac{1}{2} \\ & & & -1 & 1 \end{pmatrix}. \tag{S5}$$

Here Δx corresponds to the grid spacing in the x direction. $\mathbf{d}_{n_y}$ and $\mathbf{d}_{n_z}$ can be defined analogously. An explicit solution for Eq (S3) can be found

by enforcing Eq (S4) using appropriate Lagrange multipliers as shown in by Wang [52] (appendix 1). This results in an algorithm that starts with calculating an eigenvalue decomposition of $\mathbf{d}_{n_x}\mathbf{d}_{n_x}^T$, $\mathbf{d}_{n_y}\mathbf{d}_{n_y}^T$ and $\mathbf{d}_{n_z}\mathbf{d}_{n_z}^T$:

$$\mathbf{d}_{n_x}\mathbf{d}_{n_x}^T = \mathbf{Q}_{n_x}\mathbf{L}_{n_x}\mathbf{Q}_{n_x}^T \tag{S6}$$
$$\mathbf{d}_{n_y}\mathbf{d}_{n_y}^T = \mathbf{Q}_{n_y}\mathbf{L}_{n_y}\mathbf{Q}_{n_y}^T \tag{S7}$$
$$\mathbf{d}_{n_z}\mathbf{d}_{n_z}^T = \mathbf{Q}_{n_z}\mathbf{L}_{n_z}\mathbf{Q}_{n_z}^T \tag{S8}$$

Since each of these matrices has only the size of one of the three spatial dimensions of the sample, they are comparatively small compared to an algorithm that would target Eq (S3) directly and would have to deal with $n_x \times n_y \times n_z$ components simultaneously. Furthermore, if the sample is a square ($n_x = n_y$) or multiple data sets need to be analyzed, an already calculated eigenvalue decomposition can be reused to increase the computational efficiency.
If we now denote the components of $\mathbf{Q}_{n_x}$, $\mathbf{Q}_{n_y}$ and $\mathbf{Q}_{n_z}$ by $Q_{n_x}^{ij}$, $Q_{n_y}^{ij}$, $Q_{n_z}^{ij}$ and the diagonal elements of $\mathbf{L}_{n_x}$,$\mathbf{L}_{n_y}$ and $\mathbf{L}_{n_z}$ by $\lambda_{n_x}^k$, $\lambda_{n_y}^k$, $\lambda_{n_z}^k$, the algorithm has the following steps, that contain only basic algebra and matrix multiplications.

- Calculate the divergence residue field of the uncorrected data

$$S_{exp}^{ijk} = \sum_{i'=1}^{n_x} d_{n_x}^{ii'} u_{exp}^{i'jk} + \sum_{j'=1}^{n_y} d_{n_y}^{jj'} v_{exp}^{ij'k} + \sum_{k'=1}^{n_z} d_{n_z}^{kk'} w_{exp}^{ijk'} \tag{S9}$$

- Calculate the helper variables Γ^{lmn} given by:

$$\Gamma^{lmn} = \lambda_{n_x}^l + \lambda_{n_y}^m + \lambda_{n_z}^n \tag{S10}$$

- Calculate another set of helper variables μ^{ijk} given by:

$$\mu^{ijk} = \sum_{l,m,n=1}^{n_x,n_y,n_z} \frac{Q_{n_x}^{il} Q_{n_y}^{jm} Q_{n_z}^{kn}}{\Gamma^{lmn}} \left(\sum_{i',j',k'}^{n_x,n_y,n_z} Q_{n_x}^{i'l} Q_{n_y}^{j'm} Q_{n_z}^{k'n} S_{exp}^{i'j'k'} \right) \tag{S11}$$

  Γ^{lmn} contains a single zero element. In order to calculate μ^{ijk}, this zero element must be replaced by a non-zero value (for example one). This particular value does not affect the result for the corrected vector field. The situation is similar to the calculation of a scalar potential, where a constant offset will also have no effect on its gradient field.

- Finally the corrected field can be obtained by:

$$u_c^{ijk} = u_{exp}^{ijk} - \sum_{i'=1} d_{n_x}^{i'i} \mu^{i'jk}, \tag{S12}$$
$$v_c^{ijk} = v_{exp}^{ijk} - \sum_{j'=1} d_{n_y}^{j'j} \mu^{ij'k}, \tag{S13}$$
$$w_c^{ijk} = w_{exp}^{ijk} - \sum_{k'=1} d_{n_z}^{k'k} \mu^{ijk'}. \tag{S14}$$

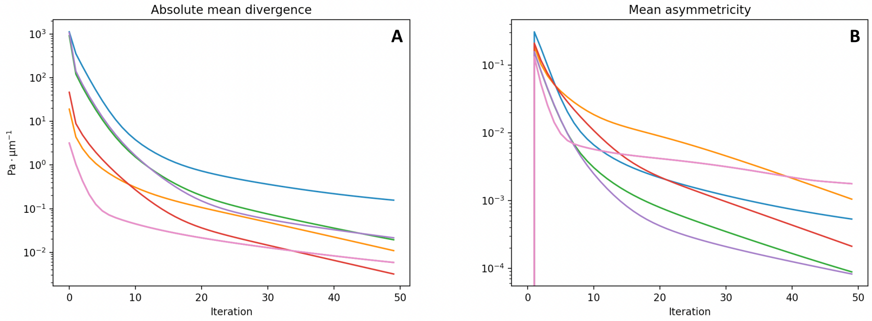

Fig S1. Absolute mean divergence of $\boldsymbol{\sigma}^{(i)}$ and mean value of the asymmetricity for $\boldsymbol{w}^{(i)}$. The blue line represents the effect of the divergence correction on random data ($\boldsymbol{\sigma}^{(0)}$ is filled with white noise.) The other lines represent data for $\boldsymbol{\sigma}^{(0)}$ obtained from different pressure and point adhesion profiles with and without noise perturbations.

S2 Convergence of the divergence correction algorithm

The iterative divergence correction scheme used for the Divergence-free Direct method 3D traction force microscopy tries to optimize the result according to two criteria:

- Minimize the divergence of $\boldsymbol{\sigma}^{(i)}$.
- Reduce the asymmetry of $\boldsymbol{w}$

We estimate the level divergence of $\boldsymbol{\sigma}^{(i)}$ by first calculating

$$S_r^{ijk} = \sum_{i'=1}^{n_x} d_{n_x}^{ii'} \sigma_{r1}^{i'jk} + \sum_{j'=1}^{n_y} d_{n_y}^{jj'} \sigma_{r2}^{ij'k} + \sum_{k'=1}^{n_z} d_{n_z}^{kk'} \sigma_{r3}^{ijk'} \tag{S15}$$

for $\sigma_{rl} = (\boldsymbol{\sigma}^{(i)})_{rl}$ for $r = 1, 2, 3$ using the first order discrete derivatives and then taking the L2 norm

$$\sum_{i,j,k} \sqrt{(S_1^{ijk})^2 + (S_2^{ijk})^2 + (S_3^{ijk})^2} \tag{S16}$$

The asymmetricity of a matrix can be described by

$$\text{asym}(\mathbf{w}) = \frac{\|\mathbf{w} - \mathbf{w}^T\|}{2\|\mathbf{w}\|} \tag{S17}$$

where $\text{asym}(\mathbf{w}) = 1$ indicates a fully asymmetric and $\text{asym}(\mathbf{w}) = 0$ a symmetric matrix.
As we can see in figure S1, every iteration improves both, the symmetricity of $\boldsymbol{u}$ as well as the the remaining divergence $\nabla\boldsymbol{\sigma}$ is reduced. However no universal termination criteria can be extracted from either improvement. Notice that the rate of improvement reduces after 5 to 20 iterations. For this reason, we assume that a sufficient level of improvement is reached after 20 iterations and terminate the divergence correction algorithm at this point.

S3 3D displacement fields for a Hertz-like force profile

We want to find the full space solution for the infinite halve space problem presented in Eq 11 for traction profile

$$\boldsymbol{\tau} = \frac{3}{2\pi a^3}\mathbf{Q}\sqrt{a^2-(x-x_0)^2-(y-y_0)^2}\,\Theta(a^2-(x-x_0)^2-(y-y_0)^2). \tag{S18}$$

We will make use of the Boussinesq-Cerruti potential functions introduced in the description of the 3D FTTC method. It is sufficient to find a solution for $x_0 = y_0 = 0$ for the special cases $\mathbf{Q} = (Q,0,0)^T$ and $\mathbf{Q} = (0,0,P)^T$. The general solution can be found by linear transformations and rotations as well as superposition.
We deal with $\mathbf{Q} = (0,0,P)^T$ first. Following an ansatz put forward by [54], we first define a function

$$\mathcal{M}(x,y,z) = \int_0^a t(\xi)k(x,y,z+i\xi)\,\mathrm{d}\xi \tag{S19}$$

Where k is chosen in a such a way that $\mathcal{M}$ is a complex harmonic function ($\nabla^2\mathcal{M} = 0$):

$$k(x,y,z_1) = \frac{1}{2}\left(z_1^2 - \frac{1}{2}r^2\right)\ln(z_1+R_1) - \frac{3}{4}R_1z_1 + \frac{1}{4}r^2. \tag{S20}$$

We used the abbreviations $r = \sqrt{x^2+y^2}$ and $R_1 = \sqrt{z_1^2+r^2}$ and $t(\xi)$ is a function defined on the integration interval. We can now find solutions to Eq 11 by setting

$$P_x = 0 \qquad P_y = 0 \qquad P_z = \operatorname{Im}\mathcal{M} \tag{S21}$$

The definitions of P_x and P_y provide trivial solutions to their boundary value problems. The field equation for P_z is already satisfied, because P_z is harmonic from being the imaginary part of a complex harmonic function. The boundary condition for P_z results in an integral problem for $t(\xi)$ given by:

$$\begin{aligned}\tau_z(x,y) = -\partial_z^3 P_z|_{z=0} &= -\operatorname{Im}\int_0^a t(\xi)\frac{1}{\sqrt{r^2+(i\xi)^2}}\,\mathrm{d}\xi \\ &= \int_r^a t(\xi)\frac{1}{\sqrt{\xi^2-r^2}}\,\mathrm{d}\xi\end{aligned}\quad. \tag{S22}$$

In case of the Hertz potential given by Eq S18 with $\mathbf{Q} = (0,0,P)^T$ this can be solved by setting

$$t(\xi) = \frac{3P}{2\pi a^3}\xi. \tag{S23}$$

To solve the resulting integral formula Eq S19, we transform the integral path onto a complex curve:

$$\begin{aligned}P_z(x,y,z) &= \operatorname{Im}\frac{3P}{2\pi a^3}\int_\gamma (z-z_1)k(x,y,z_1)\,\mathrm{d}z_1 \\ &\text{for } \gamma : \xi \in [0,a] \longmapsto z_1(\xi) = z+i\xi.\end{aligned} \tag{S24}$$

We can now make use of the fact that the integrated function of this expression is holomorphic except if $z_1 = im$ for $m \in \mathbb{R}$, $|m| > r$ and apply the fundamental theorem of calculus for holomorphic functions. This means that we can find a solution by finding an anti-derivative of the integrated function, evaluating it at $z_1 = z_2 = z + ia$ and taking the imaginary part. The contribution from the lower integral boundary can be ignored because it gives only a purely real contribution, that drops out when taking the imaginary part for the final result. This yields P_z to be the imaginary part of the following expression:

$$\begin{aligned}&\frac{1}{576}\frac{3P}{2\pi a^3} r^2 \left(16z\left(4R_2 + 9z_2\right) - 9z_2\left(R_2 + 8z_2\right)\right)\\ +&\frac{1}{576}\frac{3P}{2\pi a^3} 2z_2^2 R_2 (63z_2 - 88z)\\ +&\frac{1}{576}\frac{3P}{2\pi a^3} 3\left(3r^4 + 24r^2 z_2(z_2 - 2z) + 8z_2^3(4z - 3z_2)\right)\ln\left(R_2 + z_2\right)\end{aligned} \tag{S25}$$

Here we have used the abbreviations $z_2 = z + ia$ and $R_2 = \sqrt{z_2^2 + r^2}$. The deformation fields can now be calculated directly from the potential function Eq 17. We can determine the derivatives of P_z from Eq S25. Alternatively we apply the derivative already on equations find expressions Eq S19 to get expressions like:

$$\frac{\partial}{\partial z} P_z = \mathrm{Im} \int_0^a \frac{3P}{2\pi a^3} \xi \frac{\partial k}{\partial z_1}(x, y, z + i\xi)\, \mathrm{d}\xi \tag{S26}$$

As terms like $\frac{\partial k}{\partial z_1}$ are still harmonic, the subsequent steps of integral transformation and finding the anti-derivative can be done as described above to find analytic expressions for the derivatives of P_z and thus ultimately for $\mathbf{u}$.
For the second case $\mathbf{Q} = (Q, 0, 0)^T$, a solution to Eq 11 can be found using

$$P_x = \mathrm{Im}\,\mathcal{M} \qquad P_y = 0 \qquad P_z = 0. \tag{S27}$$

where we must now set

$$t(\xi) = \frac{3Q}{2\pi a^3}\xi \tag{S28}$$

to solve the boundary condition. The remaining steps described are now analogs to $\mathbf{Q} = (0, 0, P)^T$, except that P and P_z must be replaced by Q and P_x in all terms, including Eq S25. In the same procedure as in the $\mathbf{Q} = (0, 0, P)^T$ case, we can now find $\mathbf{u}$ using Eq 15.

S4 Parameters used for simulated profiles

In general profiles can be described by

$$\boldsymbol{\tau}(x, y) = \sum_i \boldsymbol{H}(x, y, x_i, y_i, a_i, \mathbf{F}_i) \tag{S29}$$

with the definition

$$H(x, y, x', y', a, \mathbf{F}) = \frac{3}{2\pi a^3}\mathbf{F}\sqrt{a^2 - \rho^2}\,\Theta(a - \rho) \tag{S30}$$

where $\rho = \sqrt{(x - x')^2 + (y - y')^2}$.

- Profile Fig 2, Fig 4 C, Fig 5, Fig 6 C and Fig 7

Substrate properties		
Young's Modulus	E	10 kPa
Poisson Ratio	ν	0.5
Simulation properties		
Spacing x and y direction	d_{xy}	0.4 µm
Spacing z direction	d_z	0.8 µm
Number of sampling points x and y direction	n_{xy}	128
Number of sampling points z direction	n_z	6

Profile parameters						
i	x'	y'	a	F_x	F_y	F_z
1	0.0 µm	0.0 µm	6.0 µm	0.0 nN	0.0 nN	300 nN
2	−10.8 µm	6.2 µm	2.0 µm	86.6 nN	−50.0 nN	−50.0 nN
3	10.8 µm	−6.2 µm	2.0 µm	−86.6 nN	50 nN	−50.0 nN
4	7.7 µm	12.5 µm	2.0 µm	0.0 nN	−100 nN	−50.0 nN
5	−7.7 µm	−12.5 µm	2.0 µm	0.0 nN	100 nN	−50.0 nN
6	10.8 µm	6.2 µm	2.0 µm	−86.6 nN	−50.0 nN	−50.0 nN
7	−10.8 µm	−6.2 µm	2.0 µm	86.6 nN	50 nN	−50.0 nN

- Profile Fig 3 A and Fig 4 A

$$\tau_x = 0 \qquad \tau_y = 0 \qquad \tau_z = \frac{3F}{2\pi a^3}\sqrt{a^2 - r^2}\,\Theta(a-r) \tag{S31}$$

Substrate properties		
Young's Modulus	E	10 kPa
Poisson Ratio	ν	0.45
Force profile parameters		
Total Force	F	10 µN
Radius of force affect region	a	20 µm
Simulation properties		
Spacing x and y direction	d_{xy}	0.8 µm
Spacing z direction	d_z	2.0 µm
Number of sampling points x and y direction	n_{xy}	128
Number of sampling points z direction	n_z	10

- Profile Fig 3 B and Fig 4 B

$$\tau_x = 0 \quad \tau_y = 0$$
$$\tau_z = F\left(\frac{3}{2\pi a_I^3}\sqrt{a_I^2 - r^2}\,\Theta(a_I - r) - \frac{3}{2\pi a_O^3}\sqrt{a_O^2 - r^2}\,\Theta(a_O - r)\right) \tag{S32}$$

Substrate properties		
Young's Modulus	E	3 kPa
Poisson Ratio	ν	0.45
Force profile parameters		
Force amplitude	F	1 µN
Radius of force affect region	a_O	60 µm
Radius of inner region	a_I	45 µm
Simulation properties		
Spacing x and y direction	d_{xy}	0.8 µm
Spacing z direction	d_z	2.0 µm
Number of sampling points x and y direction	n_{xy}	256
Number of sampling points z direction	n_z	10

- Profile Fig 6 A

Substrate properties		
Young's Modulus	E	10 kPa
Poisson Ratio	ν	0.5
Simulation properties		
Spacing x and y direction	d_{xy}	0.4 µm
Spacing z direction	d_z	0.4 µm
Number of sampling points x and y direction	n_{xy}	128
Number of sampling points z direction	n_z	6

Profile parameters						
i	x'	y'	a	F_x	F_y	F_z
1	0.0 µm	0.0 µm	6.0 µm	0.0 nN	0 nN	100 nN
2	4.3 µm	11.7 µm	2.0 µm	−34.2 nN	−93 nN	−25 nN
3	4.3 µm	−11.7 µm	2.0 µm	−34.2 nN	93 nN	−25 nN

- Profile Fig 6 Center

Substrate properties		
Young's Modulus	E	10 kPa
Poisson Ratio	ν	0.45
Simulation properties		
Spacing x and y direction	d_{xy}	0.8 µm
Spacing z direction	d_z	4.0 µm
Number of sampling points x and y direction	n_{xy}	64
Number of sampling points z direction	n_z	4

Profile parameters						
i	x'	y'	a	F_x	F_y	F_z
1	−6.4 µm	7.7 µm	2.0 µm	128.5 nN	−153.2 nN	0.0 nN
2	6.4 µm	−7.7 µm	2.0 µm	−128.5 nN	153.2 nN	0.0 nN
3	6.1 µm	10.0 µm	2.0 µm	0 nN	−50.0 nN	0.0 nN
4	−6.1 µm	−10.0 µm	2.0 µm	0 nN	−50.0 nN	0.0 nN
5	6.4 µm	7.7 µm	2.0 µm	−64.3 nN	−76.6 nN	0.0 nN
6	−6.4 µm	−7.7 µm	2.0 µm	64.3 nN	76.6 nN	0.0 nN

S5 Effect of sampling density in combination with displacement noise

As complement to Fig 7 of the main text, which was calculated without noise, here we document the effect of changing sampling density in the presence of displacement noise. In Fig S2 and Fig S3 we simulated the effects of variation in sampling density for a high ($\sigma_N / < \|u\| >= 1$) and very high ($\sigma_N / < \|u\| >= 2$) noise level, respectively. Surprisingly, we see that the SNR of FTTC now significantly improves when decreasing the sample density (increasing the sample distance). This is due to the fact that while for the DM only neighboring points are used to calculate the local traction, in FTTC even far away points contribute. This means that increasing the sample distance decreases the number of sampling points and as such the number of points that contribute error to the error in traction at each sampling point.

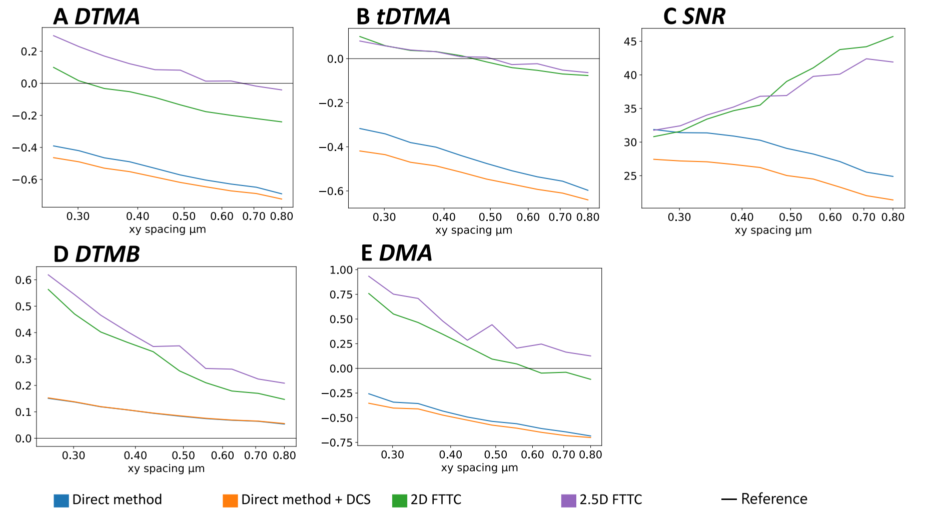

Fig S2. Effects of variation in sampling density for a high noise level. Plots A to E show how the different metrics are affected when using a different distance of the sampling points when setting up the input while adding noise with a standard deviation that is equivalent to the of the average of the mean deformation field of the unperturbed profile ($\sigma_N / < \|u\| >= 1$). Moving to higher levels of noise worsens the performance of the Fourier-based methods, while the performance of the direct method is affected less.

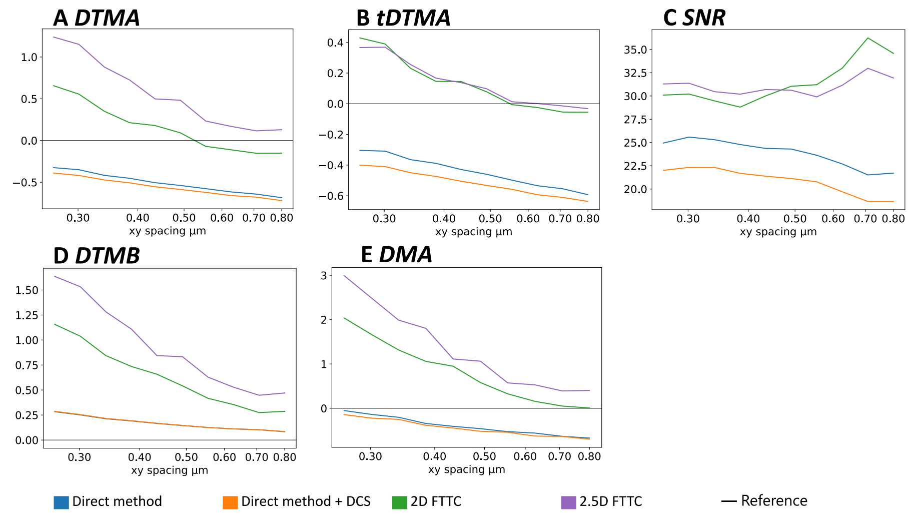

Fig S3. Effects of variation in sampling density for a very high noise level. Plots A to E show how the different metrics are affected when using a different distance of the sampling points when setting up the input while adding noise that is equivalent to twice of the average of the mean deformation field of the unperturbed profile ($\sigma_N / < \|u\| >= 2$).
